# SIRT1 Mediates the Antagonism of Wnt/β-Catenin Pathway by Vitamin D in Colon Carcinoma Cells

**DOI:** 10.7150/ijbs.95875

**Published:** 2024-10-07

**Authors:** José Manuel García-Martínez, Ana Chocarro-Calvo, Javier Martínez-Useros, Nerea Regueira-Acebedo, María Jesús Fernández-Aceñero, Alberto Muñoz, María Jesús Larriba, Custodia García-Jiménez

**Affiliations:** 1 Physiology Area, Department of Basic Health Sciences. Health Sciences Faculty, University Rey Juan Carlos, Alcorcón, Madrid, Spain.; 2 Translational Oncology Division, OncoHealth Institute, Health Research Institute-University Hospital Fundación Jiménez Díaz-Universidad Autónoma de Madrid, Spain.; 3 Department of Surgical Pathology, Hospital Clínico San Carlos, Madrid, Spain.; 4 Instituto de Investigaciones Βiomédicas Sols-Morreale, Consejo Superior de Investigaciones Científicas, Universidad Autónoma de Madrid, Madrid, Spain.; 5 Centro de Investigación Βiomédica en Red de Cáncer (CIΒERONC), Madrid, Spain.; 6 Instituto de Investigación Sanitaria del Hospital Universitario La Paz (IdiPAZ), Madrid, Spain.

**Keywords:** colorectal cancer, vitamin D, SIRT1, β-catenin, Wnt, glucose, acetylation

## Abstract

Cancer initiation and progression result from genetic and epigenetic alterations caused by interactions between environmental and endogenous factors leading to aberrant cell signalling. Colorectal cancers (CRC) are linked to abnormal activation of the Wnt/β-catenin pathway, whose key feature is the nuclear accumulation of acetylated β-catenin in colon epithelial cells. Nuclear β-catenin acts as a transcriptional co-activator, targeting genes involved in cell proliferation and invasion. 1α,25-Dihydroxyvitamin D_3_ (1,25(OH)_2_D_3_ or calcitriol), the active form of vitamin D, antagonizes Wnt/β-catenin over-activation by engaging its high affinity receptor, VDR. Here we unveil that 1,25(OH)_2_D_3_-bound VDR activates Silent Information Regulator of Transcription, sirtuin 1 (SIRT1), leading to β-catenin deacetylation and nuclear exclusion, downregulation of its pro-tumourigenic target genes and inhibition of human colon carcinoma cell proliferation. Notably, orthogonal SIRT1 activation mimics nuclear exclusion of β-catenin while SIRT1 inhibition blocks the effects of 1,25(OH)_2_D_3_. Thus, SIRT1 emerges as a crucial mediator in the protective action of vitamin D against CRC. The mutual negative feedback loop unveiled here between Wnt and SIRT1 represents an important surrogate target in CRC. Since nuclear localisation of β-catenin is a critical driver of CRC that requires its acetylation, we provide a mechanistic foundation for the epidemiological evidence linking vitamin D deficiency and increased CRC risk and mortality.

## Introduction

Epidemiological studies suggest that vitamin D (cholecalciferol) deficiency may be a risk factor for developing and dying of cancer, particularly for colon/colorectal cancer (CRC) 1-4. However, data from supplementation studies in the human population are controversial, and confirmation of a clinically relevant anti-CRC effect of vitamin D in well-designed prospective randomized trials is pending 5-9. Post-hoc analyses indicate that this discrepancy may rely on the lack of patient stratification in the clinical trials, suggesting that vitamin D intervention is effective in the deficient/insufficient vitamin D subjects but not in vitamin D sufficient individuals, and is also dependent on conditions such as patient body mass, ethnicity, mutational status, or genotype of vitamin D-related genes 6,10,11. Importantly, many mechanistic experimental studies show a wide range of anti-tumoural effects of vitamin D in CRC and other neoplasia that strongly support a protective vitamin D action 12,13.

Vitamin D is incorporated in humans through diet or is synthesized in the skin by solar ultraviolet radiation-dependent transformation of 7-dehydrocholesterol. The active vitamin D metabolite is 1α,25-dihydroxyvitamin D_3_ (1,25(OH)_2_D_3_ or calcitriol), which results from two consecutive hydroxylations of vitamin D, the first in the liver and the second in the kidney or in many epithelial and immune cell types in the organism 1,14. 1,25(OH)_2_D_3_ binds to a member of the nuclear receptor superfamily of transcription factors, the vitamin D receptor (VDR). Upon ligand binding, VDR regulates the pleiotropic actions of vitamin D including its many anticancer effects on cell survival, proliferation and differentiation 1,13-16.

The anti-CRC properties of liganded VDR largely rely on its capacity to interfere with a well-recognized CRC driver, the canonical Wnt/β-catenin signalling pathway that regulates many pro-tumoural genes and which is abnormally over-activated in most CRC 17. VDR expression is the main determinant of cell responsiveness to 1,25(OH)_2_D_3_ and is often downregulated in advanced CRC, which together with frequent vitamin D deficiency implies that these patients would probably not benefit from the anticancer effects of 1,25(OH)_2_D_3_ 18-20. Consistently, in a mouse model that predisposes for intestinal adenoma, *Apc*^Min/+^ (where *Apc* stands for *Adenomatous Polyposis Coli* and^ Min^ for Multiple Intestinal Neoplasia), germline *Vdr* deletion increases Wnt/β-catenin signalling and intestinal tumour load 21,22.

From the many possible steps to block Wnt/β-catenin signalling, the most precise is acting directly on β-catenin, its downstream transcriptional effector. Acetylation of β-catenin promotes its nuclear localisation and transcriptional activity in cancer cells 23-25. β-catenin acetylation is increased in CRC cells by the combination of Wnt signalling and enhanced glucose uptake 23-25. The coincidence of Wnt3A and high glucose increases the levels and activity of the acetyl transferase EP300 and inhibits Silent Information Regulator of Transcription, sirtuin 1 (SIRT1)-driven deacetylation of β-catenin without changing SIRT1 levels 23 by mechanisms that remain unknown. Interestingly, 1,25(OH)_2_D_3_ induces SIRT1 deacetylase activity including its auto-deacetylation in CRC cells 26.

The opposite effects of Wnt signalling and 1,25(OH)_2_D_3_ on the deacetylase activity of SIRT1 23,26 prompted us to study whether Wnt-driven inhibition of SIRT1 is mediated through acetylation and can be reversed by 1,25(OH)_2_D_3_. We also investigated whether 1,25(OH)_2_D_3_ interferes the Wnt/β-catenin pathway via regulation of SIRT1. We specifically examined the intriguing possibility that 1,25(OH)_2_D_3_ impacts on CRC by blocking the glucose-driven β-catenin acetylation that enhances Wnt/β-catenin signalling 22,27.

## Materials and Methods

Key materials and resources are listed in Supplementary Table ST1.

Clinicopathological characteristics of CRC patients included in the study are listed in Supplementary Table ST2.

### Colorectal cancer cells

Human colorectal adenocarcinoma HT-29 and HCT 116 cells and HCT 116-derived ShControl and ShVDR cells were cultured in 5% CO_2_ at 37ºC with DMEM containing 25 mM glucose supplemented with 10% foetal bovine serum (FBS) and 1% Penicillin-Streptomycin. Cells were treated as indicated for 24 h. ShControl cells and ShVDR cells were derived from HCT 116 cells as reported by Larriba *et al.* 22. Briefly, ShVDR cells are stably depleted of VDR using a shRNA targeting VDR and shControl cells stably express a non-targeting shRNA that activates the RISC complex and the RNA interference pathway but that contains at least five mismatched nucleotides compared with any human gene.

### Transient transfections

For plasmid transfection, cells were seeded in plates at 50% confluence and then transfected using JetPei PolyPlus reagent (see Supplementary Table ST1), following the manufacturer's instructions. After 24 h, cells were treated as indicated for another 24 h and then harvested for subsequent analyses.

For SIRT1 depletion, cells were plated in six well plates at 50% density and transfected with siRNAs specific for SIRT1 (see Supplementary Table ST1) using JetPRIME and following the manufacturer's instructions. Two days post-transfection, cells were treated with 40 mM LiCl for 24 h and then with 100 nM 1,25(OH)_2_D_3_ or vehicle for additional 24 h. Next, cells were harvested for western blot analysis.

### Preparation of cell extracts

#### Whole cell extracts

Cells were washed in iced-cold Phosphate-buffered saline (PBS) and scraped off in radioimmunoprecipitation assay (RIPA) buffer (10 mM Tris HCl [pH 7.4], 5 mM Ethylene diamine tetra acetic acid (EDTA), 5 mM egtazic acid (EGTA), 1% Triton X100, 10 mM Na_4_P_2_O_7_ [pH 7.4], 10 mM NaF, 130 mM NaCl, 0.1% SDS, 0.5% Na-deoxycholate, and protease inhibitor cocktail (Roche, Cat# 04693132001, Supplementary Table ST1). After 5 min on ice, cell debris was pelleted by centrifugation at 13,500 *g* for 5 min at 4ºC, and the supernatant was used as whole cell extract.

#### Fractionated cell extracts

Cells were washed as before and scraped off in hypotonic buffer (20 mM Hydroxy ethyl piperazine ethane sulfonic acid (Hepes) [pH 8.0], 10 mM KCl, 0.15 mM EDTA, 0.15 mM EGTA, 0.05% Nonidet P-40 (NP40), and protease inhibitor cocktail as explained above for whole cell extracts) and incubated on ice for 10 min before adding 1:2 vol. of sucrose buffer (50 mM Hepes [pH 8.0], 0.25 mM EDTA, 10 mM KCl, and 70% sucrose). Lysates were fractionated by centrifugation at 2,370 *g* for 5 min at 4ºC to obtain the cytoplasmic fraction in the supernatant. Nuclear pellets were washed twice with washing buffer (20 mM Hepes [pH 8.0], 50 mM NaCl, 1.5 mM MgCl_2_, 0.25 mM EDTA, 0.15 mM EGTA, 25% glycerol, and protease inhibitor cocktail), resuspended in nuclear extraction buffer (20 mM Hepes [pH 8.0], 450 mM NaCl, 1.5 mM MgCl_2_, 0.25 mM EDTA, 0.15 mM EGTA, 0.05% NP40, 25% glycerol, and protease inhibitor cocktail), and centrifuged at 13,500 *g* for 5 min at 4ºC to pellet and discard cell debris. The supernatants were used as nuclear fractions.

### Immunoprecipitation

Nuclear extracts (300 µg) were diluted 1/10 in immunoprecipitation buffer (20 mM Hepes [pH 8.0], 10 mM KCl, 0.15 mM EDTA, 0.15 mM EGTA, 50 mM NaCl, 0.05% Nonidet P-40 (NP40) and protease inhibitor cocktail). Protein A/G-coated magnetic beads (Invitrogen) were prepared by washing 3 times in immunoprecipitation buffer and coating them with the primary antibody (0.1-1.0 µg of antibody) by incubation at 4ºC in a rotating wheel for 2 h, followed by elimination of unbound antibody by washing twice in the same buffer. The beads were then incubated with the diluted nuclear extracts overnight at 4ºC in a rotating wheel. Immunocomplexes were recovered by applying a magnetic field, washed twice in immunoprecipitation buffer and then used for western blotting.

### Western blotting

Lysates or immunoprecipitates were denatured and loaded on sodium dodecyl sulfate polyacrylamide gels and then transferred to polyvinylidene difluoride membranes (Bio-Rad). After blocking with 5% (w/v) Bovine Serum Albumin (BSA) or nonfat dry milk the membrane was incubated with the corresponding primary and HRP-conjugated secondary antibodies. The specific bands were analysed using Typhoon scanner control 3.0 or ChemiDoc MP Imaging Systems (Bio-Rad) and quantified using Image J software.

### Immunofluorescence

Cells on coverslips were washed three times in PBS, fixed with 4% paraformaldehyde in PBS [pH 7.4] for 10 min, washed again, and permeabilized for 5 min using a buffer containing PBS [pH 7.4], 0.5% Triton X-100, and 0.2% BSA. Nonspecific binding was blocked by adding PBS [pH7.4], 0.05% Triton X-100, and 5% BSA for 1 h at room temperature before incubation with primary antibody overnight at 4ºC. To remove unbound antibody, coverslips were washed three times in PBS for 5 min at room temperature. Then, they were incubated with fluorophore-conjugated secondary antibody for 1 h at room temperature, washed and incubated for 10 min at room temperature with a 1/10000 dilution in PBS of DAPI (4',6-diamidino-2-phenylindole, dihydrochloride) from Invitrogen. Slides were mounted, and images were acquired using a SP5 confocal microscope (Leica) or FV3000 confocal microscope (Olympus) with a 63X objective. Fluorescence intensity was quantified using Image J software. For each experiment, three different fields were evaluated per slide.

### Cell growth curves

Cells seeded at a density of 20,000 cells per well in Corning 12-well plates were treated according to the experiment for 1 to 6 days. For colorimetric determination of growth, cells were treated with 3-(4,5-dimethylthiazol-2-yl)-2,5-diphenyltetrazolium bromide (MTT) (Sigma-Aldrich) in culture medium (1:10 ratio) and incubated at 37°C for 3 h. In living cells, MTT is reduced to formazan, which has a purple colour. Then, the medium was removed, and the formazan was solubilized in dimethylsulfoxide (DMSO), transferred to 96-well plates and measured by a Spectra FLUOR (Tecan) at 542 nm. Cell viability was analysed in four independent experiments.

### RT-qPCR

Total RNA was extracted from cells using TRIzol reagent (Invitrogen). One μg of total RNA was reverse transcribed using the High-Capacity Reverse Transcription kit (Applied Biosystems, see Supplementary Table ST1) and then subjected to qPCR amplification using the master mix kits: PowerTrack™ SYBR Green and TaqMan™ Fast Advanced and a 7500 Fast Real-Time PCR System, both kits and equipment from Applied Biosystems; the primers and TaqMan probes are indicated in Supplementary Table ST1. Ribosomal 18S RNA expression served as a control. Relative expression was calculated using the Ct method, expressed as 2^-ΔΔCt^ 28. PCR efficiency was close to 100%.

### Human samples

Colorectal cancer patient samples were obtained from surgical resections of 149 patients diagnosed with CRC at stage II or at stage IV with liver metastases at the Fundación Jiménez Díaz University Hospital (Madrid, Spain). These samples were used to set up tissue microarrays and a database with patient clinicopathological information.

### Ethics approval and consent to participate

This study was reviewed and approved by the Institutional Review Board (IRB) of the Fundación Jimenez Diaz Hospital on December 9, 2014 (Act number 17/14). CRC tissues were collected using protocols approved by the corresponding Ethics Committees following the Spanish legislation. All patients gave written informed consent for the use of their biological samples for research purposes. Fundamental ethical principles and rights promoted by Spain (LOPD 15/1999) and the European Union (2000/C364/01) were followed. Patient data were processed according to the Declaration of Helsinki (last revision 2013) and Spanish National Biomedical Research Law (14/2007, July 3).

### Tissue microarrays and immunohistochemistry

Tissue microarrays (TMA) containing samples (n = 149) from primary tumours of stage II (n = 95) and liver metastases of stage IV (n = 54) CRC patients were constructed using the MTA-1 tissue arrayer (Beecher Instruments, Sun Prairie) for immunohistochemistry analysis. Each sample (diameter 1 mm) was punched from pre-selected tumour regions in paraffin-embedded tissues. Tumour central areas avoiding necrosis foci were chosen. Staining was conducted in 2 μm sections. As many as 81/95 samples from stage II and 26/54 samples from stage IV were considered optimal for both histological and immunohistochemical evaluation. Slides were deparaffinized by incubation at 60°C for 10 min and then incubated with PT-Link (Dako, Agilent) for 20 min at 95°C at pH 6.0 to detect SIRT1, AceH3K9, AceH4 and Acep53; pH 8.0 to detect β-catenin. Slides in slide-holders were incubated with peroxidase blocking reagent (Dako, Agilent) and then with the following dilutions of antibodies: anti-SIRT1 (1:50), anti-Acep53 (1:100), anti-AceH4 (1:50) and anti-AceH3K9 (1:50), overnight at 4°C and anti-β-catenin (1:500) for 20 min at room temperature. Slides were incubated for 20 min with the appropriate HRP-conjugated anti-Ig (EnVision, Dako, Agilent). Sections were then visualized with 3,3'-diaminobenzidine (Dako, Agilent) as a chromogen for SIRT1 and β-catenin or with the HRP Magenta (Dako, Agilent) for AceH3K9, AceH4 and Acep53, and counterstained for 5 min with Harrys' Haematoxylin (Sigma Aldrich, Merck). Photographs were taken with a stereo microscope (Leica DMi1). According to the *The Human Protein Atlas* (available at http://www.proteinatlas.org, accessed on July 2024), a human testis tissue was used as a positive control for anti-SIRT1, a human colonic tissue was used for both anti-AceH3K9 and anti-AceH4, and a human kidney tissue for anti-Acep53. Immunoreactivity was quantified with an Histoscore (H score, range 0-300) that considers both the intensity (low, medium, or high) and the percentage of positively stained cells following this algorithm: H score = (low %) × 1 + (medium %) × 2 + (high %) × 3. Quantification for each patient biopsy was calculated blindly by 2 investigators (MJFA and JMU). AceH3K9, AceH4 and SIRT1 showed nuclear staining, whereas β-catenin and Acep53 were in the nucleus and cytoplasm. Clinicopathological characteristics of the patients are summarized in Supplementary Table ST2.

### Statistical analyses

For immunohistochemistry data, to determine whether each of the antigens evaluated were well-modelled by a normal distribution, Kolmogorov-Smirnov test was used. All factors showed non-parametric distributions. Therefore, analyses between groups (stage II tumours versus stage IV metastases) were performed with Mann-Whitney U-test and correlations with Spearman test (S). Association analysis between SIRT1 protein levels and cytoplasmic β-catenin protein levels was performed with Chi-square test, considering median of SIRT1 H-score and median of cytoplasmic β-catenin H-score as cut-off points to separate samples between high- or low- expression levels.

For the rest of the data, parametric analysis between two sample groups were performed with Student's t test, and for multiple comparisons ANOVA with Bonferroni's post-test was used.

All statistical analyses were performed with SPSS IBM software v.24. P-values < 0.05 were considered statistically significant.

## Results

### 1,25(OH)_2_D_3_ reverses the acetylation of SIRT1 promoted by Wnt and induces β-catenin nuclear export in CRC cells

**Figure 1:**
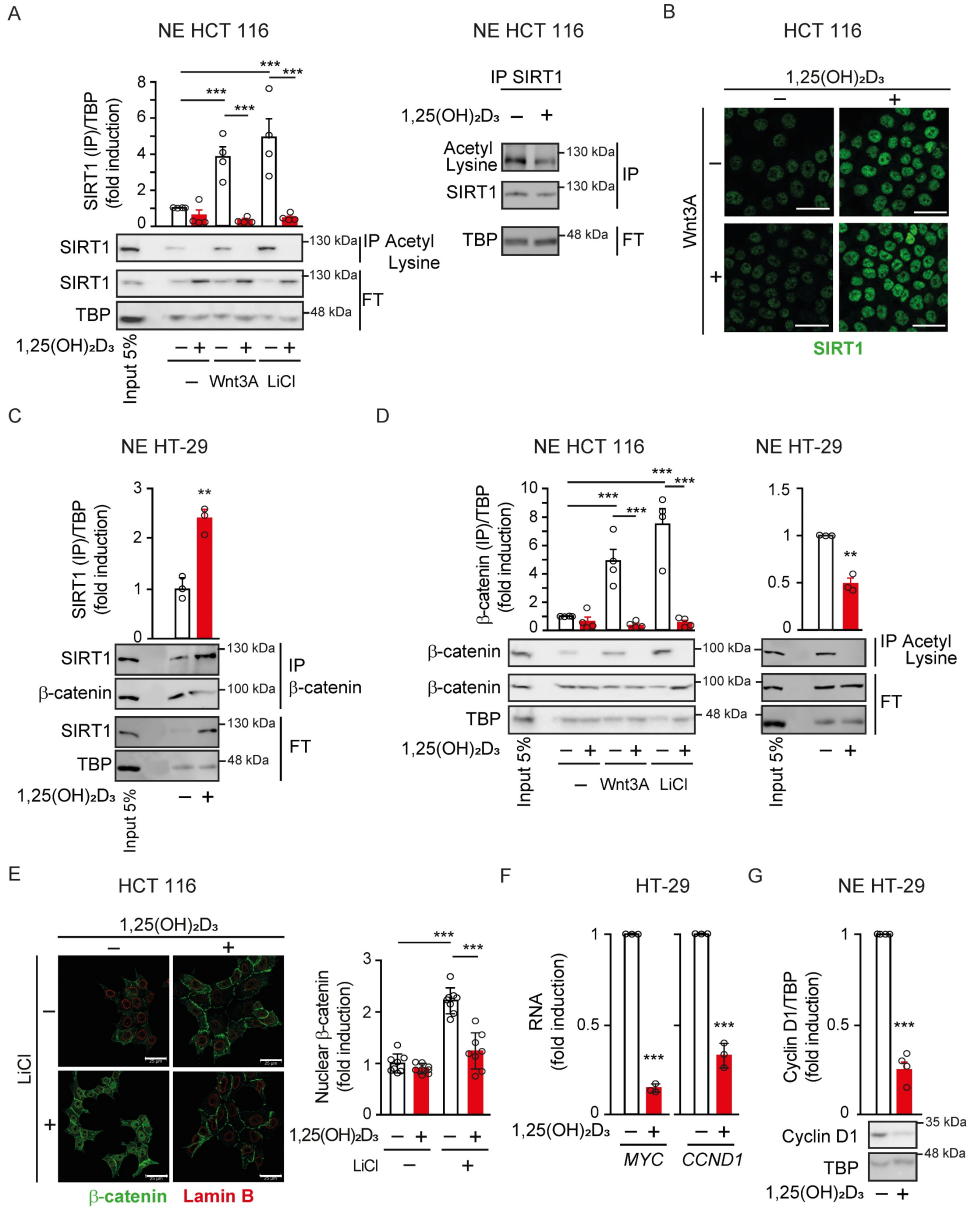
** 1,25(OH)_2_D_3_ reverses Wnt-induced nuclear β-catenin acetylation, accumulation and transcriptional effects *via* the increase in SIRT1 expression and deacetylase activity.** (A) Western blot analysis of the effects of 1,25(OH)_2_D_3_ on the acetylation of SIRT1 protein in the presence or absence of Wnt3A or LiCl. Nuclear extracts (NE) from HCT 116 cells treated with vehicle, Wnt3A (100 ng/ml) or LiCl (40 mM) for 24 h and then with vehicle or 1,25(OH)_2_D_3_ (100 nM) for additional 24 h were immunoprecipitated (IP) using anti-acetyl-lysine (left) or anti-SIRT1 antibodies (right). Representative blots and statistical analysis using TBP in the flow through (FT) as loading control. (B) Confocal imaging analysis of the effect of 1,25(OH)_2_D_3_ on SIRT1 expression in HCT 116 CRC cells cultured in the presence or absence of Wnt3A for 24 h. Scale bars: 25 µm. (C) 1,25(OH)_2_D_3_ effects on the SIRT1-β-catenin interaction in the nuclei of HT-29 cells. Immunoprecipitates using anti-SIRT1 antibody were subsequently analysed by western blotting using anti-β-catenin antibody. Input (5%) and flow through (FT) are shown. TBP served as loading control of the IP and western blot. (D) Western blot analysis of the effects of 1,25(OH)_2_D_3_ on the acetylation of β-catenin protein induced by Wnt3A or LiCl as indicated in (A). Nuclear extracts (NE) from HCT 116 cells (left) or HT-29 cells (right) were immunoprecipitated (IP) upon 24 h treatment using anti-acetyl-lysine antibody and the immunoprecipitates were subsequently analysed by western blotting using anti-β-catenin (left panel) or immunoprecipitated with anti-β-catenin antibody and analysed by western blotting using anti-acetyl-lysine (right panel). Representative blots and statistical analysis using TBP in the flow through (FT) as control. (E) Left, confocal imaging analysis of the effect of 1,25(OH)_2_D_3_ on the localisation of β-catenin protein (green) in HCT 116 cells treated or not with 1,25(OH)_2_D_3_ for 24 h. Nuclear envelope was marked using anti-Lamin B antibody (red). Scale bars: 25 µm. Right, quantification of fluorescence intensity in 3 independent experiments using ImageJ software; 2-3 different fields were evaluated per slide. (F)-(G) Effect of 1,25(OH)_2_D_3_ (100 nM, 24 h) on the expression of Wnt target genes in HT-29 cells. (F) RT-qPCR analysis of the RNA level of *MYC* and *CCND1*. Values were normalized to those of 18S RNA level and are referred as fold induction over vehicle-treated cells. (G) Western blot analysis of cyclin D1 protein level in nuclear extracts of HT-29 cells. A representative blot is shown. TBP was used as loading control. The full distribution of the data is shown. For western blots (panels A, C, D and G) mean ± SEM of replicates is displayed to illustrate the representability of the western blot shown. For immunofluorescence quantification and RT-qPCR (panels E-F) mean ± standard deviation (SD) is presented to show data variability. Statistical analysis by Student t-test of at least 3 independent experiments was performed; *P < 0.05; **P < 0.01; ***P < 0.001.

The acetylation status of SIRT1 in the nuclei of CRC cells was studied by exposure of cells to Wnt3A or to LiCl, a glycogen synthase kinase inhibitor that mimics Wnt signalling. Notably, Wnt signalling strongly induced SIRT1 acetylation that was reversed by treatment with 1,25(OH)_2_D_3_ (Figure 1A); similar results were obtained by immunoprecipitation of acetyl-lysine and immunoblot with anti-SIRT1 (Figure 1A, left) or *vice versa* (Figure 1A, right). Additionally, 1,25(OH)_2_D_3_ increased SIRT1 levels in CRC cells independently of the presence of Wnt signals (Figure 1Β; see nuclear staining with DAPI as control in Supplementary S1A). 1,25(OH)_2_D_3_ induction of SIRT1 levels was not restricted to CRC cells and took place in other unrelated cancer cell lines from pancreas or melanoma (Supplementary Figure S1B) suggesting that this might be a general mechanism for 1,25(OH)_2_D_3_ action. Thus, 1,25(OH)_2_D_3_ ensures SIRT1 induction by increasing both its level and its deacetylation. Interestingly, co-immunoprecipitation assays demonstrated that 1,25(OH)_2_D_3_ favoured SIRT1-β-catenin interaction (Figure 1C). Consistently, 1,25(OH)_2_D_3_ promoted β-catenin deacetylation in HCT 116 and HT-29 colon carcinoma cells (Figure 1D and Supplementary S1C), which paralleled β-catenin nuclear exclusion as examined by western blotting (Supplementary Figure S1D) and by immunofluorescence (Figure 1E and Supplementary Figure S1E). Accordingly, 1,25(OH)_2_D_3_ reduced the expression of β-catenin target genes associated with tumour progression such as *MYC* (Myelocytomatosis) and *CCND1* (encoding Cyclin D1) (Figure 1F, and Supplementary Figure S1F). In addition, 1,25(OH)_2_D_3_ also reduced the protein levels of the critical cell cycle regulator Cyclin D1 (Figure 1G). SIRT1 induction by 1,25(OH)_2_D_3_ was specific since other nuclear deacetylases such as SIRT7 or HDAC1 remain unchanged (Supplementary Figure S1G). Notably, 1,25(OH)_2_D_3_ could interfere with Wnt signalling independently of whether it was added before or after Wnt activation (Supplementary Figure S1H), suggesting a potential for both prevention and treatment.

### SIRT1 protein expression directly correlates with the level of cytoplasmic β-catenin and SIRT1 deacetylase activity declines along human CRC progression

To confirm whether the link between β-catenin localisation and SIRT1 expression and deacetylase activity also takes place in human CRC, we next analysed β-catenin subcellular distribution and SIRT1 level and activity in tissue microarrays (TMA) containing samples (n = 107) from colorectal primary tumours of stage II patients and liver metastases of stage IV patients. SIRT1 deacetylase activity was inferred by evaluating the acetylation of several of its well-characterized substrates such as lysine 9 of histone 3 (AceH3K9) 29, lysines 5, 8, 12 and 16 of histone 4 (AceH4) 30, and lysine 382 of p53 (Acep53) 31**.** To verify that these proteins are SIRT1 targets, changes in their acetylation status after modulation of SIRT1 activity were followed in HCT 116 CRC cells. Western blot data show that SIRT1 inhibition using EX527 (Selisistat) increased basal acetylation levels of these substrates (Supplementary Figure S2A-C), whereas specific SIRT1 activation using the small molecule SRT1720 reduced their acetylation and blocked the effect of 1,25(OH)_2_D_3._ Consistently, overexpression of SIRT1 wild type (WT) or mutants constitutively active (K610R) or catalytically dead (H363Y) respectively abolished or maintained the acetylation of H4 and p53 (Supplementary Figure S2D).

**Figure 2:**
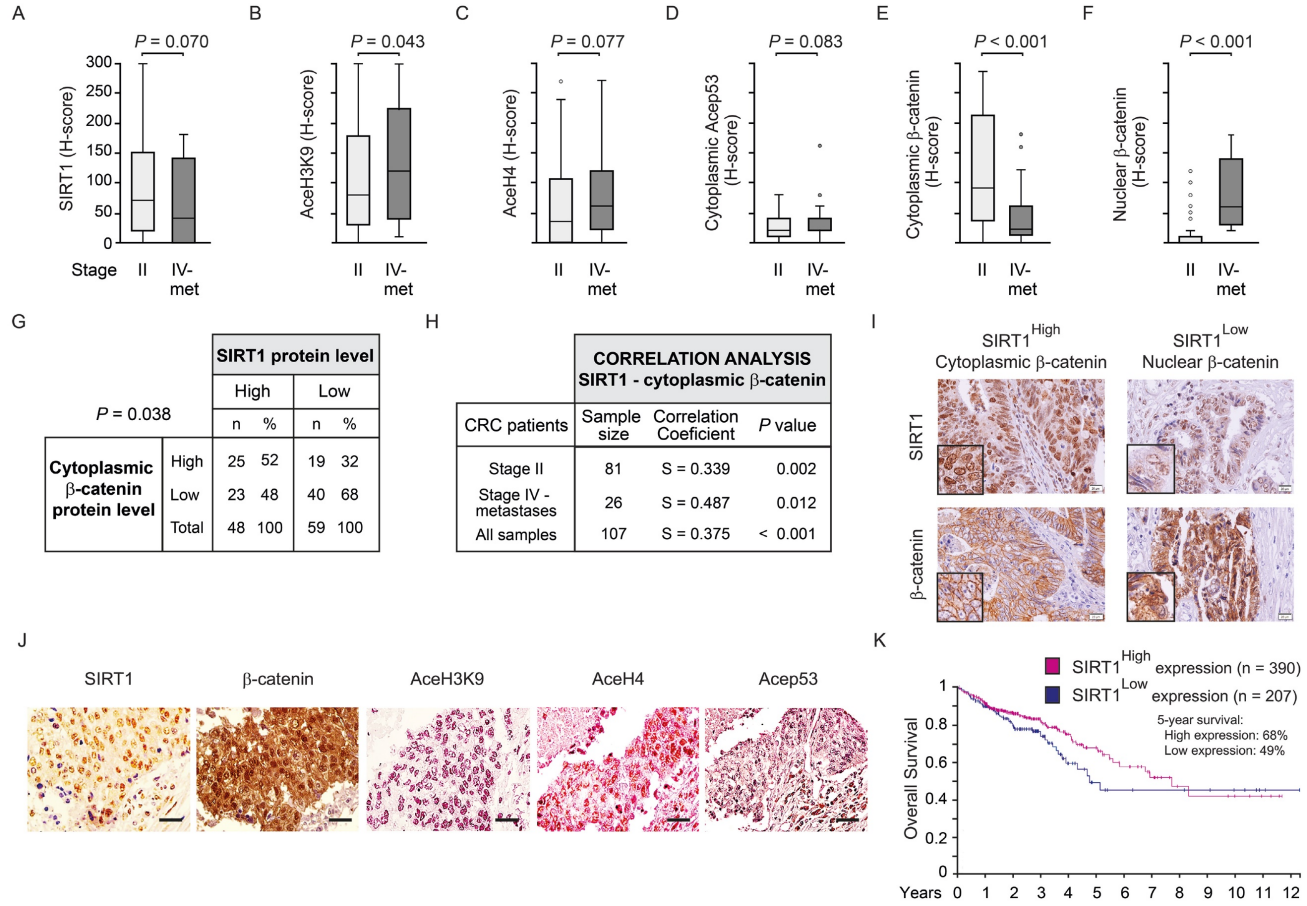
** Inverse correlation between cellular SIRT1 deacetylase activity and nuclear β-catenin content during human CRC progression.** (A)-(F) Box plots corresponding to the expression (estimated as Histo-score, H-score) in CRC stage II primary tumours and stage IV liver metastasis (met) of: (A) SIRT1, (B) AceH3K9, (C) AceH4 (K5,8,12,16), (D) Acep53 (K382) (E) cytoplasmic β-catenin and (F) nuclear β-catenin. Values represent median ± SD. The Y axis scale is the same for panels A to F. (G) SIRT1 and cytoplasmic β-catenin protein levels (graded as high or low) (H-scores) in CRC stage II and stage IV liver metastasis. (H) Correlation analysis between the levels of SIRT1 and that of nuclear or cytoplasmic β-catenin proteins in CRC patient biopsies of stage II and/or stage IV liver metastases. (I) Representative micrographs (40X) from immunostainings for SIRT1 and β-catenin proteins. A tumour sample from a patient with high SIRT1 (left) and another with low SIRT1 (right) are shown. Inserts show areas at high magnification. (J) Representative micrographs (40X) from immunostainings for SIRT1 (brown) and its target substrates β-catenin (brown), aceH3K9 (magenta), aceH4 (magenta) and AceP53 (magenta), and counterstained with haematoxylin (blue) (same CRC patient). (K) Kaplan-Meier curves performed using the best cut-off point and data from 597 CRC patients from the *The Human Protein Atlas*; SIRT^High^ (n = 390); SIRT^Low^ (n = 207). Scale bars: 20 µm. *P* < 0.05.

Quantification of SIRT1 protein levels in the TMAs revealed a high trend towards a decrease in metastases compared with stage II primary tumours (*P* = 0.070) (Figure 2A). The level of AceH3K9, as a mark for lack of SIRT1 activity, increased significantly (*P* = 0.043) from primary tumours of stage II patients to metastases (Figure 2B). Likewise, the alternative surrogates for SIRT1 inactivity showed clear trends to increase from stage II primary tumours to metastasis: AceH4 (*P* = 0.077) (Fig 2C) and Acep53 (K382) (*P* = 0.083) (Fig 2D). Accordingly, the expression of cytoplasmic and nuclear β-catenin was respectively reduced and increased along tumour progression with very high statistical significance (*P* < 0.001) (Figure 2E and 2F).

Interestingly, a significant positive association (*P* = 0.038) (Figure 2G) and correlation (Spearman coefficient = 0.375; *P* < 0.001) (Figure 2H) between the levels of SIRT1 and cytoplasmic β-catenin proteins were found in CRC samples. In addition, significant positive correlations between these parameters were also observed considering separately primary tumours from stage II patients (Spearman coefficient = 0.339, *P* = 0.002), and liver metastases (Spearman coefficient = 0.487, *P* = 0.012) (Figure 2H). Thus, human CRC samples with low levels of SIRT1 (SIRT1^Low^) tend to have high level of nuclear β-catenin and *vice versa*, as shown in representative images from consecutive sections (Figure 2I). Samples with high SIRT1 level (SIRT1^High^) tend to have high cytoplasmic β-catenin, although some SIRT1^High^ samples exhibited nuclear β-catenin, suggesting that SIRT1 deacetylase activity was diminished. This was supported by the high acetylation of SIRT1 substrates AceH3K9, AceH4 (K5, 8, 12, 16) and Acep53 (K382) in these samples (Figure 2J). Additional tumour images of consecutive sections with high or low SIRT1 level as well as example images for β-catenin quantification cut-offs are shown in Supplementary Figure S2E and S2F, respectively.

In line with these results, analysis of 597 patients from the *The Human Protein Atlas* (accessed July 2024) revealed no significant changes in overall survival but a trend (*P* = 0.091) towards significance for increased survival of patients with high SIRT1 during the first 8 follow-up years (Figure 2K). Collectively, these results suggest that decreased SIRT1 deacetylase activity drives a failure to export β-catenin from the nucleus and may worsen CRC prognosis.

### SIRT1 is required for nuclear exclusion of β-catenin and Wnt target gene inhibition by 1,25(OH)_2_D_3_

**Figure 3:**
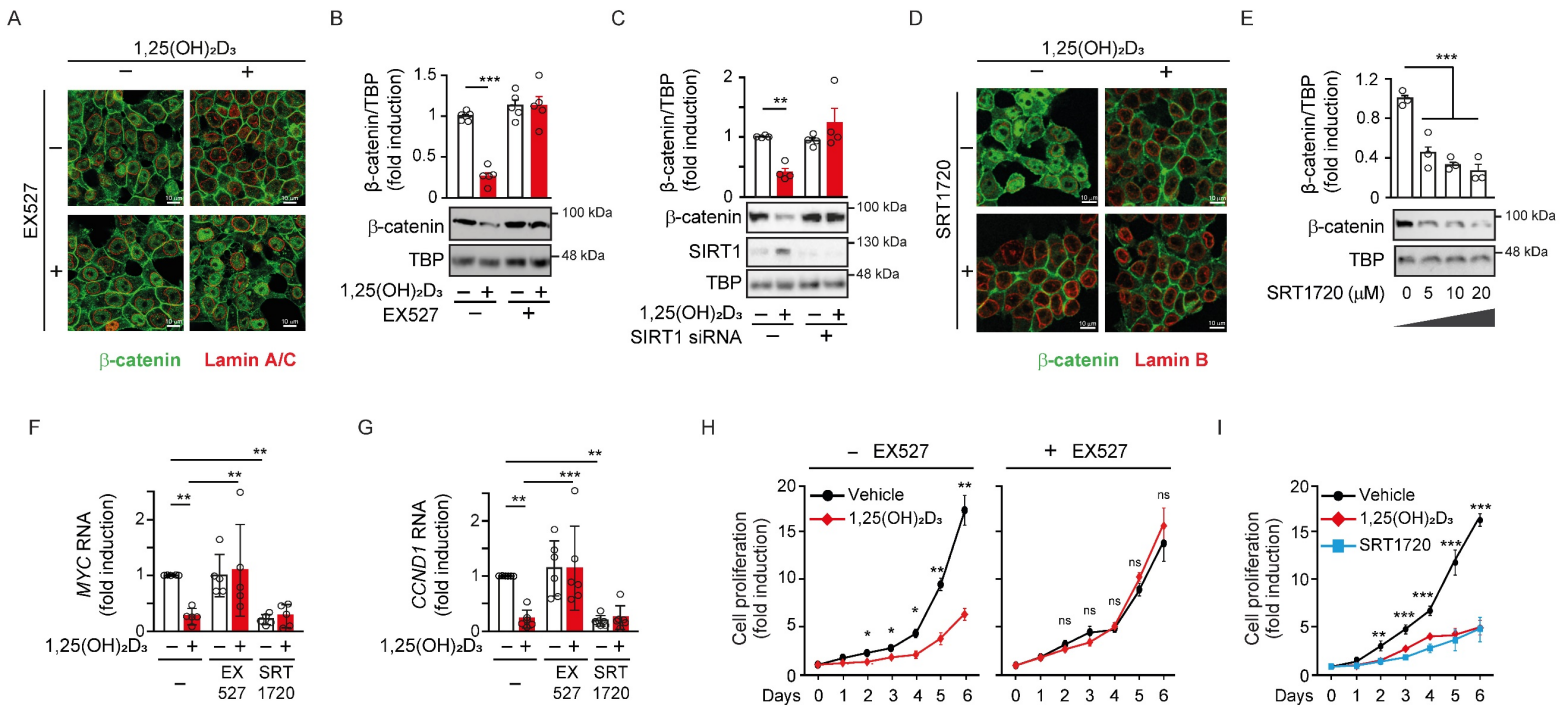
** SIRT1 is required for nuclear exclusion of β-catenin protein by 1,25(OH)_2_D_3_.** HCT 116 CRC cells were cultured under standard conditions with LiCl (40 mM) to mimic Wnt signal and then treated as indicated. (A)-(B) Effects of 1,25(OH)_2_D_3_ and the SIRT1 inhibitor EX527 (10 μM) on β-catenin subcellular localisation in HCT 116 cells. (A) Confocal immunofluorescence imaging of β-catenin (green) and Lamin A/C (red), which marks the nuclear envelope. Scale bars: 25 µm. (B) Representative western blot and statistical analysis of at least 3 independent experiments showing the level of β-catenin in nuclear extracts of HCT 116 cells treated with 1,25(OH)_2_D_3_ and/or EX527 as indicated. TBP was used as control. (C) SIRT1 depletion abolishes the reduction of nuclear β-catenin level by 1,25(OH)_2_D_3_. Representative western blot and statistical analysis of at least 3 independent experiments showing the level of β-catenin in nuclear extracts of HCT 116 cells transfected with SIRT1 siRNA or control siRNA (-) and treated with vehicle or 1,25(OH)_2_D_3_ as indicated. TBP was used as control. (D)-(E) Effects of 1,25(OH)_2_D_3_ and SIRT1720 on β-catenin subcellular localisation in HCT 116 cells. (D) Confocal immunofluorescence imaging of β-catenin (green) and Lamin B (red), which marks the nuclear envelope. Scale bars: 25 µm. (B) Representative western blot and statistical analysis of 3 independent experiments showing the level of β-catenin in nuclear extracts of HCT 116 cells treated with the indicated doses of SRT1720 for 24 h. TBP was used as control. (F)-(G) RT-qPCR analysis of the effect of 1,25(OH)_2_D_3_ in the presence or absence of the SIRT1 deacetylase activity modulators EX527 (10 μM) and SRT1720 (10 μM) on the expression of the Wnt target genes *MYC* (F) and *CCND1* (G). (H) EX527 (10 μM) blocks the 1,25(OH)_2_D_3_-driven inhibition of HCT 116 cell proliferation. (I) 1,25(OH)_2_D_3_ (100 nM) and SRT1720 (10 μM) have comparable inhibitory effect on HCT 116 cell proliferation. The full distribution of the data is shown. For western blotting (panels B, C and E) mean ± SEM of replicates is displayed to illustrate the representability of the western blot shown. For RT-qPCR (panels F-G) mean ± SD is presented to show the variability of the experiments. Statistical analysis by Student t-test (panels B, C and H) or ANOVA (panels E, G and I) of 3 independent experiments; *P < 0.05; **P < 0.01; ***P < 0.001.

To understand whether the nuclear depletion of β-catenin induced by 1,25(OH)_2_D_3_ was caused by SIRT1 induction, we modulated SIRT1 deacetylase activity. Treatment with the specific SIRT1 inhibitor EX527 prevented the nuclear depletion of β-catenin induced by 1,25(OH)_2_D_3_ in CRC cells as analysed by immunofluorescence (Figure 3A) and western blotting (Figure 3Β). Also, the general sirtuin inhibitor nicotinamide (NAA) reproduced this result (Supplementary Figure S3A-S3B). Moreover, SIRT1 depletion using several siRNAs (Supplementary Figure S3C) also blocked the 1,25(OH)_2_D_3_-mediated nuclear depletion of β-catenin (Figure 3C and Supplementary Figure S3D). Conversely, specific activation of SIRT1 using SRT1720 drove nuclear exclusion of β-catenin mimicking the effect of 1,25(OH)_2_D_3_, as shown by immunofluorescence (Figure 3D) and western blotting (Figure 3E).

Consistent with previous results, the downregulation of Wnt/β-catenin target genes critical for cell proliferation such as *MYC* or *CCND1* by 1,25(OH)_2_D_3_ was blocked by SIRT1 inhibition using EX527 and, conversely, reproduced by SIRT1 activation using SRT1720 (Figure 3F and 3G). Likewise, the expression of other Wnt/β-catenin target genes, such as the prototype *AXIN2,* were similarly up- or down-regulated by EX527 or SRT1720 driven modulation of SIRT1 activity, while the inhibition of *AXIN2* gene expression by 1,25(OH)_2_D_3_ was interfered by SIRT1 activity modulation (Supplementary Figure S3E).

The expression of other WNT target, Dickkopf WNT signalling pathway inhibitor 1 (DKK1) gene, was also analysed. Importantly, *DKK1 is* also targeted by 1,25(OH)_2_D_3_ through alternative mechanisms 32,33. The upregulation of *DKK1* by 1,25(OH)_2_D_3_ was prevented by EX527, while activation of SIRT1 with SRT1720 increased *DKK1* gene expression and allowed further induction by 1,25(OH)_2_D_3_ (Supplementary Figure S3F). Of note, the level of Myc and Cyclin D1 proteins changed according to RNA expression (Supplementary Figure S3G-H). Thus, the blockade of Wnt/β-catenin target gene expression by 1,25(OH)_2_D_3_ was impeded by SIRT1 inhibition and was reproduced by SIRT1 induction. In line with these results, SIRT1 inhibition with EX527 abolished the antiproliferative effect of 1,25(OH)_2_D_3_ on CRC cells (Figure 3H), while similar antiproliferative effects were obtained by activation of SIRT1 with either 1,25(OH)_2_D_3_ or SRT1720 (Figure 3I). Together, these results show that the 1,25(OH)_2_D_3_-driven depletion of nuclear β-catenin that interferes Wnt signalling is critically mediated by SIRT1 deacetylase activity.

### A constitutively active SIRT1 mutant mimics the reduction of nuclear β-catenin by 1,25(OH)_2_D_3_

**Figure 4:**
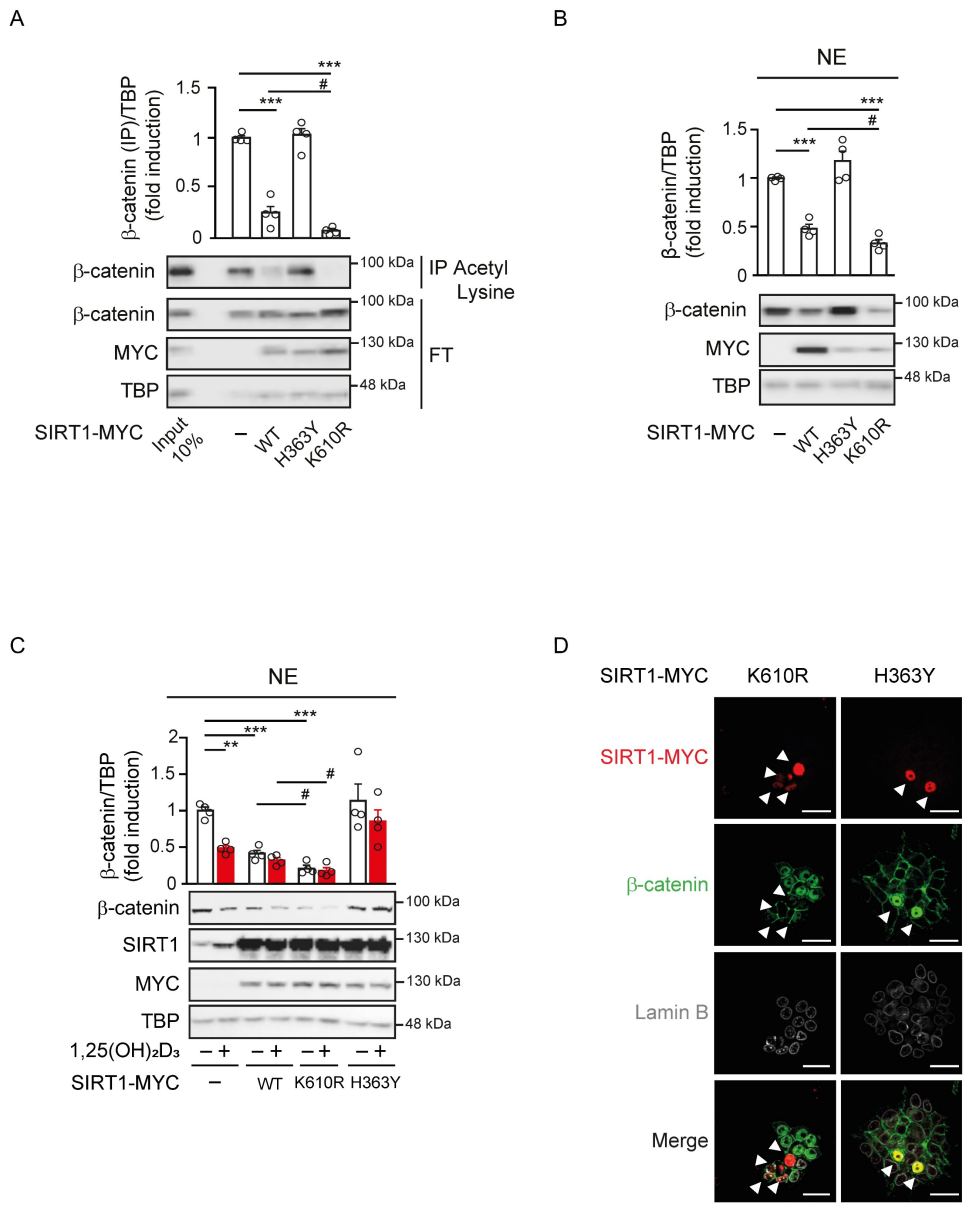
** Constitutively SIRT1 activation excludes β-catenin from the cell nucleus.** (A)-(B) Effect of wild type (WT) or mutant SIRT1 on β-catenin acetylation and nuclear accumulation. (A) Analysis of the level of acetylated nuclear β-catenin in HCT 116 cells 48 h after transfection with Myc-tagged WT SIRT1 or inactive H363Y SIRT mutant or constitutive active K610R SIRT1 mutant. Nuclear extracts (NE) were immunoprecipitated with anti-acetyl-lysine antibody and immunoprecipitates subjected to western blot analysis using anti-β-catenin antibody. FT, flow through. (B) Western blot analysis of the effect of exogenous expression of WT or the two mutant SIRT1 on nuclear β-catenin content. (C) Modulation of 1,25(OH)_2_D_3_ effect on nuclear β-catenin content by WT and mutant SIRT1. Representative western blot of the level of β-catenin protein in nuclear extracts (NE) of HCT 116 cells transfected with the Myc-tagged WT or mutant SIRT1 forms and treated at 24 h post transfection with vehicle or 1,25(OH)_2_D_3_ (100 nM) for 24 h more. In (A)-(C) antibodies against Myc and TBP were respectively used as transfection and loading controls. (D) Confocal imaging of the effects of exogenous expression of active or inactive SIRT1 mutants on nuclear β-catenin location. Arrowheads mark transfected cells expressing the SIRT1 mutants. Antibodies against Myc-tagged SIRT1 mutants (red), β-catenin (green) and Lamin B to mark the nuclear envelope (white) were used. Bottom panels show merge immunostainings. Scale bars, 25 μm. The full distribution of the data is shown. Mean ± SEM of replicates is displayed to illustrate the representability of the western blot shown. Statistical analysis by One Way ANOVA of at least 3 independent experiments; *P < 0.05; **P < 0.01; ***P < 0.001. Specific pairs of variables were additionally compared using Student t-test and presented as (#); #*P* < 0.05.

Given the role of SIRT1 on the subcellular localisation of β-catenin, we exogenously expressed SIRT1 to ask whether it mediates 1,25(OH)_2_D_3_ action controlling β-catenin acetylation and localisation. To this end, Myc-tagged wild-type (WT) SIRT1, or the inactive H363Y 31 or the constitutively active K610R 26 SIRT1 mutants were expressed to similar levels in CRC cells. Nuclear extracts from cells transfected with WT or mutant SIRT1 were immunoprecipitated using anti-acetyl-lysine antibodies and subjected to western blotting to evaluate the extent of β-catenin acetylation. In the nuclei of CRC cells, acetylated β-catenin decreased upon expression of WT SIRT1, did not significantly change after expression of the inactive H363Y SIRT1 mutant and was lost upon expression of the constitutively active K610R SIRT1 mutant (Figure 4A). Accordingly, the nuclear level of β-catenin reflected its acetylation status and was reduced by half after expression of WT SIRT1, whereas was unaffected by the inactive H363Y SIRT1, and was almost completely absent (4-5-fold reduction) following expression of the constitutively active K610R SIRT1 (Figure 4Β).

We also examined the subcellular localisation of β-catenin in response to 1,25(OH)_2_D_3_ upon expression of these mutants. 1,25(OH)_2_D_3_ treatment in control cells reduced the level of nuclear β-catenin up to 50%, whereas in cells exogenously expressing WT or the constitutively active K610R SIRT1 nuclear β-catenin diminished respectively by 40% or 75% regardless of 1,25(OH)_2_D_3_ treatment (Figure 4C). Conversely, cells expressing the inactive H363Y SIRT1 mutant exhibited higher basal nuclear level of β-catenin that were unresponsive to 1,25(OH)_2_D_3_ (Figure 4C). Of note, immunofluorescence analyses confirmed that K610R SIRT1 was constitutively active and depleted β-catenin from the nucleus of HCT 116 cells, while, in contrast, the inactive H363Y SIRT1 mutant led to nuclear accumulation of β-catenin (Figure 4D). Collectively, our data indicated that 1,25(OH)_2_D_3_ induces SIRT1 deacetylase activity, which controls β-catenin acetylation, subcellular localisation, and transcriptional activity.

### SIRT1 deacetylase activity offers a surrogate target to inhibit the Wnt/β-catenin pathway in cases of 1,25(OH)_2_D_3_ unresponsiveness

**Figure 5:**
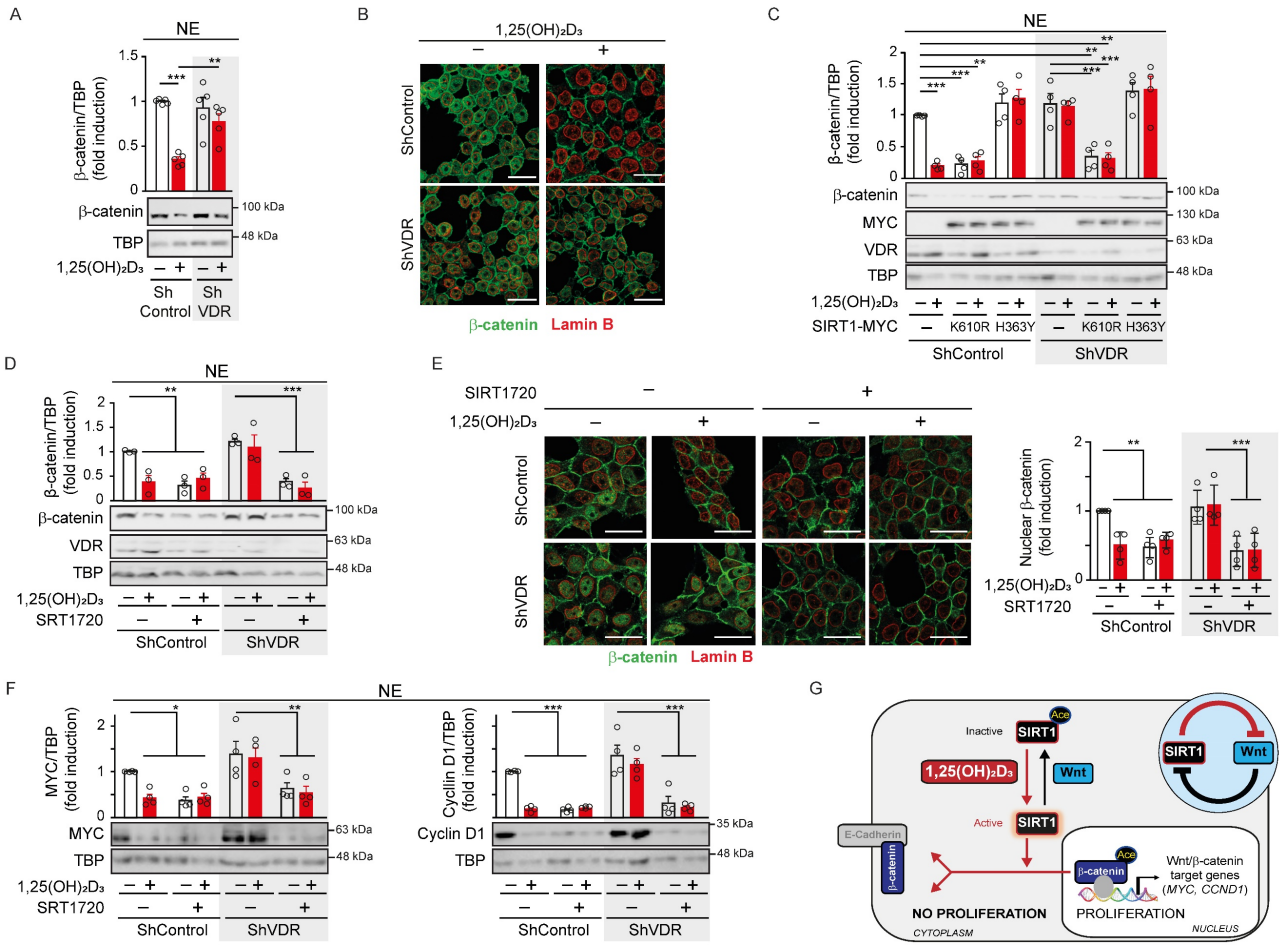
** SIRT1 deacetylase activity is a surrogate marker for vitamin D action in colon carcinoma cells.** (A) Western blot analysis of β-catenin in nuclear extracts (NE) of HCT 116-derived ShVDR or ShControl cells upon 24 h treatment with 1,25(OH)_2_D_3_ or vehicle. TBP was used as control. (B) Confocal immunostaining imaging of the effect of 1,25(OH)_2_D_3_ (100 nM, 24 h) on β-catenin (green) subcellular localisation in HCT 116 cells. Lamin B (red) marks the nuclear envelope. Scale bars, 25 μm. (C) Effect of the exogenous expression of Myc-tagged mutant H363Y or K610R SIRT1 on the nuclear exclusion of β-catenin protein induced by 1,25(OH)_2_D_3_. Representative western blot and statistical analyses; the levels of VDR and mutant SIRT1 were detected using anti-VDR and anti-Myc antibodies. TBP was used as loading control. (D) Western blot and statistical analyses of nuclear β-catenin content in HCT 116-derived ShVDR or ShControl cells treated with 1,25(OH)_2_D_3_ (100 nM) or SIRT 1720 (20 μM) for 24 h. (E) Left, confocal immunostaining imaging of the expression of β-catenin (green) in HCT 116-derived ShVDR or ShControl cells treated with 1,25(OH)_2_D_3_ (100 nM) or SRT 1720 (10 μM) for 24 h. Lamin B (red) marks the nuclear envelop. Scale bars, 25 μm. Right: quantification of nuclear β-catenin content and statistical analysis of at least 3 independent experiments. (F) Western blots and statistical analyses of the nuclear level of the Wnt targets Myc and Cyclin D1 proteins in HCT 116-derived ShVDR or ShControl cells treated with 1,25(OH)_2_D_3_ (100 nM) or SRT1720 (10 μM) for 24 h. TBP as loading control. (G) Scheme showing of the novel 1,25(OH)_2_D_3_-driven mechanism to interfere Wnt/β-catenin signalling in colon carcinoma cells based on the results of this study. Over-activation of Wnt/β-catenin signalling caused by common mutations in CRC promotes SIRT1 acetylation and inactivation, which maintains key residues of β-catenin protein acetylated. This favours β-catenin nuclear location and transcriptional activation of many target genes involved in cell proliferation and invasion. This situation is reversed by 1,25(OH)_2_D_3_ by inducing SIRT1 deacetylase activity which results in deacetylation and nuclear exclusion of β-catenin and so, the repression of Wnt/β-catenin target genes and pro-tumoural effects. The circular inset summarizes the mutual inhibition between Wnt and SIRT1. The full distribution of the data is shown. As in previous figures, mean ± SEM of replicates is displayed for western blots (panels A, C, D and F), and median ± SD for immunofluorescence quantification (panel E). Statistical analysis by One-Way ANOVA of 3 independent experiments was performed; *P < 0.05; **P < 0.01; ***P < 0.001.

VDR expression is the main determinant of cell responsiveness to 1,25(OH)_2_D_3_ and is often downregulated in advanced CRC, which together with frequent vitamin D deficiency, implies that these patients would probably not benefit from the anticancer effects of 1,25(OH)_2_D_3_ 18-20. We sought to investigate the potential of targeting SIRT1 under these conditions by using HCT 116 cells in which VDR expression was downregulated by means of shRNA 22. Western blotting and immunofluorescence analyses showed that ShControl cells responded to 1,25(OH)_2_D_3_ with strong nuclear β-catenin depletion, while ShVDR cells did not (Figure 5A-B).

Next, we examined the potential to overcome the lack of response to 1,25(OH)_2_D_3_ in ShVDR HCT 116 cells by expressing SIRT1 mutants (Figure 5C). Interestingly, expression of the constitutively active K610R SIRT1 mutant reduced nuclear β-catenin levels in both ShControl and ShVDR cells to a similar extent to that achieved by 1,25(OH)_2_D_3_ treatment in ShControl cells. As expected, the inactive H363Y SIRT1 mutant did not modify nuclear β-catenin levels (Figure 5C). These results suggested that activating SIRT1 deacetylase activity might alleviate the effects of vitamin D deficiency or unresponsiveness. Indeed, the specific SIRT1 activator SRT1720 reduced nuclear β-catenin levels in both ShControl and ShVDR cells, as shown in nuclear extracts by western blotting (Figure 5D) or in whole cells by immunofluorescence analysis (Figure 5E). Accordingly, SRT1720 reduced the levels of the Wnt/β-catenin target proteins Myc and Cyclin D1 in ShControl cells at a comparable magnitude to 1,25(OH)_2_D_3._ Notably, SRT1720 also reduced Myc and Cyclin D1 protein levels in vitamin D unresponsive ShVDR cells (Figure 5F). These results indicate that Wnt/β-catenin signalling can be reduced by activating SIRT1 in cells unresponsive to 1,25(OH)_2_D_3_.

Altogether, this work demonstrates that 1,25(OH)_2_D_3_ (i) induces reversal of SIRT1 acetylation imposed by Wnt, (ii) favours SIRT1/β-catenin interaction, (iii) increases β-catenin deacetylation, (iv) promotes nuclear exclusion of β-catenin and (v) decreases expression of key Wnt/β-catenin target genes (Figure 5G).

## Discussion

Here we reveal that SIRT1 deacetylase activity is a key mediator of the anti-CRC properties of 1,25(OH)_2_D_3_ related to its interference of Wnt/β-catenin signalling. The critical importance of Wnt signalling in CRC is emphasized by the fact that over 94% of primary and up to 96% of metastatic colorectal tumours contain mutations that aberrantly activate Wnt/β-catenin signalling 34,35. Importantly, Wnt/β-catenin pathway induces a gene signature (largely coincident with that of intestinal stem cells) that identifies CRC stem cells and predicts disease relapse 36.

Previous work of our group and others has unveiled several actions by which 1,25(OH)_2_D_3_ interferes Wnt/β-catenin signalling, such as VDR-dependent increased E-cadherin expression and improved E-cadherin-β-catenin interactions at the plasma membrane 37, competition between VDR and Tcf/Lef transcription factors to bind β-catenin 12,13,38, and the induction of the Wnt inhibitor DKK-1 33. This work demonstrates that inhibition or depletion of SIRT1 hampers the ability of 1,25(OH)_2_D_3_ to interfere Wnt/β-catenin target gene expression, and also the proliferation in CRC cells, thus providing a novel and crucial mechanism of the anti-CRC action of 1,25(OH)_2_D_3_.

Acetylation governs protein-protein and protein-DNA interactions due to increased size of the lysine side chain and neutralization of its positive charge, potentially disrupting salt bridges and introducing steric bulks 39-41. Indeed, VDR deacetylation by SIRT1 increases its transcriptional activity on specific genes 42 and may as well drive increased expression of alternative VDR target genes capable to interfere Wnt/β-catenin signalling such as DKK1. Moreover, SIRT1-driven deacetylation of β-catenin may establish the mark for its preference to bind VDR instead of Tcf/Lef in the nucleus, its capacity to be exported and its interaction with E-cadherin at the plasma membrane. Not surprisingly, there is an increasing interest on the effects of 1,25(OH)_2_D_3_ on SIRT1 43, and on the modulation by 1,25(OH)_2_D_3_ of VDR and SIRT1 interactions with FoxO and NF-κB as mediators of 1,25(OH)_2_D_3_ pleiotropism 43-45. Furthermore, our results suggest that the induction of SIRT1 by 1,25(OH)_2_D_3_ might be a general mechanism which also occurs in melanoma and pancreatic cancer. In addition, other nuclear receptors interact with SIRT1 at different levels 46,47 and the outcomes of the interplay between SIRT1 and nuclear receptors are an attractive field that warrants further exploration.

The role of SIRT1 in cancer is controversial and complicated given the multiplicity of its interactions with histones, transcriptional regulators and enzymes and its control of genome stability, cellular differentiation, growth, and metabolism 48,49. Although Liu *et al.* 50 reported that SIRT1 is overexpressed in certain tumours, suggesting that SIRT1 acts as tumour promoter, Wang *et al.* 51 reported that SIRT1-deficient embryos exhibit genomic instability and increased tumourigenesis, and Firestein *et al.* 52 showed that SIRT1 suppressed tumour growth in a mouse CRC model driven by Wnt/β-catenin. This last model suggested that the tumour suppressive action of SIRT1 must be exerted on Wnt/β-catenin. Notably, SIRT1 levels and activity are not regulated in parallel and there are CRCs with high SIRT1 levels and low SIRT1 deacetylase activity 26. Therefore, reports on increased SIRT1 levels in CRC through tumour progression, for example 53, do not demonstrate that SIRT1 deacetylase activity also increases, especially in the presence of Wnt signalling, which we show that inactivates SIRT1. This is also important to interpret the correlation between SIRT1 overexpression or depletion and tumour size in xenografts 53, since increased SIRT1 levels may not correlate with increased SIRT1 deacetylase activity. Intriguingly, SIRT1 levels were recently reported to correlate with decreased overall survival 54 using a TMA with 90 CRC patient samples, which is in clear contrast with our results and with the Human Protein Atlas database. Whether this is due to differences between the populations of patients analysed, types of treatment, diet or other lifestyle variables needs further exploration.

Importantly, by using the specific SIRT1 activator SRT1720, the SIRT1 inhibitors NAA and EX527, several siRNAs against SIRT1, and overexpression of SIRT1 WT and constitutively active K610R and inactive H363Y mutants, we convincingly show that the depletion of nuclear β-catenin and the antiproliferative effects exerted by 1,25(OH)_2_D_3_ in CRC cells rely on SIRT1 induction and subsequent β-catenin deacetylation. Notably, we also show that the reported inactivation of SIRT1 by Wnt signalling 23,24 correlates with SIRT1 acetylation that is reversed by 1,25(OH)_2_D_3_ treatment, reported to activate SIRT1^26^.

Increased nuclear β-catenin content was found in HeLa cells upon SIRT1 overexpression 55, which led to conclude that SIRT1 deacetylation of β-catenin promotes its nuclear entry. This is at odds with the opposite phenotype reported in this work and with the established general assumption that β-catenin acetylation improves its stability by inhibiting its ubiquitin-mediated degradation and promotes its nuclear translocation 23,56-59. Nonetheless, HeLa cells under basal conditions showed abundant SIRT1 with most β-catenin in the cytosol 53. In contrast to this study, our work shows unequivocally that cells expressing constitutively active SIRT1 K610R mutant exhibit nuclei lacking β-catenin, whereas cells expressing the inactive H363Y mutant accumulate nuclear β-catenin. In addition, we show nuclear β-catenin depletion and deacetylation following SIRT1 activation or overexpression. Nude mice with HeLa xenografts overexpressing SIRT1 were described to develop bigger tumours 55 but this could be achieved independently of Wnt/β-catenin signalling given the pleiotropic effects of SIRT1 on global transcription, genome stability, and metabolism. Even the interaction of SIRT1 with Wnt signalling occurs at multiple levels including the downregulation of Dvl 60 and silencing of Wnt antagonists such as DACT1 and SFRPs 61. In this context, future studies should examine xenograft tumours of *SIRT1* KO HCT 116 cells exposed to 1,25(OH)_2_D_3_.

Interestingly, SIRT1-driven deacetylation and nuclear exclusion of β-catenin in HCT 116 cells was also recently reported, which is in agreement with our results 54. Remarkably this study focused on deacetylation of β-catenin K49, which interferes with ubiquitination-driven proteolysis 62. However, nuclear localisation and increased transcriptional activity of β-catenin was previously reported to rely on (de)acetylation of K354 23. Importantly, several acetylation sites present in β-catenin (including K19, K49, K345, K354) and targeted by different acetyl transferases (CΒP, EP300, pCAF) govern its affinity for different proteins and may do so in a cell type specific manner. This may offer an explanation to the distinct outcomes of β-catenin deacetylation in distinct cell types.

Our results show that activation of SIRT1 by 1,25(OH)_2_D_3_ reverses SIRT1 inhibition by Wnt and leads to exclusion of β-catenin from the nuclei of CRC cells. Vitamin D deficiency is strongly associated epidemiologically to CRC but also to diabetes 12,13,63. Since the effects described here reverse the potentiation of Wnt signalling exerted by high glucose in diabetes 24,64-68, our results suggest the interesting possibility that vitamin D deficiency may be one underlying cause connecting these two diseases.

Wnt signalling regulates development and drives adult tissue stem cell maintenance. Although its malfunction is involved in cancer and many other diseases, it is therapeutically underexploited due to toxicity and limited efficacy 69,70. Unfortunately, despite the potential of 1,25(OH)_2_D_3_ to interfere Wnt/β-catenin signalling, advanced CRCs often loss VDR expression and become 1,25(OH)_2_D_3_ unresponsive. Here, we highlight that, downstream of VDR, small molecule activators for SIRT1 such as SRT1720 offer an alternative approach and a therapeutic hope for these cancers if they could be directed specifically to cancer cells. Our results point to SIRT1 as a critical effector of 1,25(OH)_2_D_3_ and rise the hope that acting on SIRT1 may circumvent 1,25(OH)_2_D_3_ unresponsiveness derived from vitamin D deficiency or VDR loss as it is common in advanced CRC. Finally, vitamin D and SIRT1 function have been recognised as critical for multiple diseases with inflammatory components 71,72, therefore, our conclusions may extend beyond cancer and suggest a potential for SIRT1 activators to alleviate the effects of vitamin D deficiency in multiple systems.

## Supplementary Material

Supplementary figures and tables.

## Abbreviations

AceH3K9: acetylated histone H3 at lysine 9
AceH4: acetylated histone H4
Acep53: acetylated Tumour suppressor p53
*Apc* ^Min/+^: mouse model where *Apc* stands for *Adenomatous Polyposis Coli* and^ Min^ for Multiple Intestinal Neoplasia
AXIN2: gene encoding the protein axis inhibition protein 2
CΒP: CREB-binding protein
CCND1: gene encoding the protein Cyclin D1
CRC: Colorectal cancer
DACT1: Dishevelled binding antagonist of β-catenin 1
DAPI: 4',6-diamidino-2-phenylindole, dihydrochloride
DMSO: dimethylsulfoxide
DMEM: Dulbecco's Modified Eagle Medium
DKK1: gene encoding the protein Dickkopf-related protein 1
EDTA: Ethylene diamine tetra acetic acid
EGTA: ethylene glycol-bis(β-aminoethyl ether)-N,N,N′,N′-tetra acetic acid) or egtazic acid
EP300: gene encoding the acetyl transferase protein p300
FBS: foetal bovine serum
FoxO: Forkhead box O transcription factors
FT: Flow through
IP: immunoprecipitated
HDAC1: gene encoding the protein histone deacetylase 1
Hepes: Hydroxy ethyl piperazine ethane sulfonic acid
IRB: Institutional Review Board
KO: Knock Out
*MYC*: gene encoding the protein Myc (Myelocytomatosis)
MTT: 3-(4,5-dimethylthiazol-2-yl)-2,5-diphenyltetrazolium bromide
NAA: Nicotinamide
NF-κB: Nuclear Factor Kappa B Subunit 1
NP40: Nonidet P40 or nonyl phenoxypolyethoxylethanol
PBS: Phosphate-buffered saline
pCAF: p300/CBP-associated factor
RIPA: radioimmunoprecipitation assay buffer
RISC: RNA-induced silencing complex
SD: Standard deviation
SEM: standard error of the mean
SFRPs: Secreted Frizzled Related Proteins
SIRT1: Silent Information Regulator of Transcription, sirtuin 1
TBP: TATA binding protein
Tcf/Lef: T cell factor/lymphoid enhancer factor transcription factors
TMA: Tissue microarrays
VDR: vitamin D receptor
WT: wild type

## References

[1] Feldman D, Krishnan A V, Swami S, Giovannucci E, Feldman BJ (2014). The role of vitamin D in reducing cancer risk and progression. Nat Rev Cancer.

[2] Grant WB, Boucher BJ, Al Anouti F, Pilz S (2022). Comparing the Evidence from Observational Studies and Randomized Controlled Trials for Nonskeletal Health Effects of Vitamin D. Nutrients.

[3] Kim H, Yuan C, Nguyen LH, Ng K, Giovannucci EL (2023). Prediagnostic Vitamin D Status and Colorectal Cancer Survival by Vitamin D Binding Protein Isoforms in US Cohorts. J Clin Endocrinol Metab.

[4] Kim H, Lipsyc-Sharf M, Zong X, Wang X, Hur J, Song M (2021). Total Vitamin D Intake and Risks of Early-Onset Colorectal Cancer and Precursors. Gastroenterology.

[5] Chandler PD, Chen WY, Ajala ON, Hazra A, Cook N, Bubes V (2020). Effect of Vitamin D _3_ Supplements on Development of Advanced Cancer. JAMA Netw Open.

[6] Henn M, Martin-Gorgojo V, Martin-Moreno JM (2022). Vitamin D in Cancer Prevention: Gaps in Current Knowledge and Room for Hope. Nutrients.

[7] Manson JE, Bassuk SS, Buring JE, VITAL Research Group (2020). Principal results of the VITamin D and OmegA-3 TriaL (VITAL) and updated meta-analyses of relevant vitamin D trials. J Steroid Biochem Mol Biol.

[8] Song M, Lee IM, Manson JAE, Buring JE, Dushkes R, Gordon D (2021). No Association Between Vitamin D Supplementation and Risk of Colorectal Adenomas or Serrated Polyps in a Randomized Trial. Clinical Gastroenterology and Hepatology.

[9] Muñoz A, Grant WB (2022). Vitamin D and Cancer: An Historical Overview of the Epidemiology and Mechanisms. Overview of the Epidemiology and Mechanisms Nutrients.

[10] Kanno K, Akutsu T, Ohdaira H, Suzuki Y, Urashima M (2023). Effect of Vitamin D Supplements on Relapse or Death in a p53-Immunoreactive Subgroup With Digestive Tract Cancer: Post Hoc Analysis of the AMATERASU Randomized Clinical Trial. JAMA Netw Open.

[11] Gwenzi T, Schrotz-King P, Schöttker B, Hoffmeister M, Brenner H (2023). Vitamin D Status, Cdx2 Genotype, and Colorectal Cancer Survival: Population-Based Patient Cohort. Nutrients 2023, Vol 15, Page 2717.

[12] Ferrer-Mayorga G, Larriba MJ, Crespo P, Muñoz A (2019). Mechanisms of action of vitamin D in colon cancer. J Steroid Biochem Mol Biol.

[13] Carlberg C, Muñoz A (2022). An update on vitamin D signaling and cancer. Semin Cancer Biol.

[14] Bikle D, Christakos S (2020). New aspects of vitamin D metabolism and action — addressing the skin as source and target. Nat Rev Endocrinol.

[15] Carlberg C, Velleuer E (2022). Vitamin D and the risk for cancer: A molecular analysis. Biochem Pharmacol.

[16] Fernández-Barral A, Bustamante-Madrid P, Ferrer-Mayorga G, Barbáchano A, Larriba MJ, Muñoz A (2020). Vitamin D Effects on Cell Differentiation and Stemness in Cancer. Cancers (Basel).

[17] González-Sancho JM, Larriba MJ, Muñoz A (2020). Wnt and vitamin d at the crossroads in solid cancer. Cancers.

[18] Ferrer-Mayorga G, Gómez-López G, Barbáchano A, Fernández-Barral A, Peña C, Pisano DG (2017). Vitamin D receptor expression and associated gene signature in tumour stromal fibroblasts predict clinical outcome in colorectal cancer. Gut.

[19] Li L, Wu B, Liu JY, Yang LB (2013). Vitamin D receptor gene polymorphisms and type 2 diabetes: A Meta-analysis. Arch Med Res.

[20] Sun J (2017). The Role of Vitamin D and Vitamin D Receptors in Colon Cancer. Clin Transl Gastroenterol.

[21] Zheng W, Wong KE, Zhang Z, Dougherty U, Mustafi R, Kong J (2012). Inactivation of the vitamin D receptor in APC(min/+) mice reveals a critical role for the vitamin D receptor in intestinal tumor growth. Int J Cancer.

[22] Larriba MJ, Ordóñez-Morán P, Chicote I, Martín-Fernández G, Puig I, Muñoz A (2011). Vitamin D receptor deficiency enhances Wnt/β-catenin signaling and tumor burden in colon cancer. PLoS One.

[23] Chocarro-Calvo A, García-Martínez JM, Ardila-González S, De la Vieja A, García-Jiménez C (2013). Glucose-Induced β-Catenin Acetylation Enhances Wnt Signaling in Cancer. Mol Cell.

[24] Garcia-Jimenez C, Garcia-Martinez JM, Chocarro-Calvo A, De la Vieja A (2013). A new link between diabetes and cancer: Enhanced WNT/β-catenin signaling by high glucose. J Mol Endocrinol.

[25] Gutiérrez-Salmerón M, García-Martínez JM, Martínez-Useros J, Fernández-Aceñero MJ, Viollet B, Olivier S (2020). Paradoxical activation of AMPK by glucose drives selective EP300 activity in colorectal cancer. PLoS Biol.

[26] García-Martínez JM, Chocarro-Calvo A, Martínez-Useros J, Fernández-Aceñero MJ, Fiuza MC, Cáceres-Rentero J (2023). Vitamin D induces SIRT1 activation through K610 deacetylation in colon cancer. Elife.

[27] Ren J-J (2023). Serum 25-hydroxyvitamin D concentrations and colorectal cancer incidence in adults with type 2 diabetes. British Journal of Cancer.

[28] Livak KJ, Schmittgen TD (2001). Analysis of relative gene expression data using real-time quantitative PCR and. Methods.

[29] Fang J, Ianni A, Smolka C, Vakhrusheva O, Nolte H, Krüger M (2017). Sirt7 promotes adipogenesis in the mouse by inhibiting autocatalytic activation of Sirt1. Proc Natl Acad Sci U S A.

[30] Bell EL, Nagamori I, Williams EO, Del Rosario AM, Bryson BD, Watson N (2014). SirT1 is required in the male germ cell for differentiation and fecundity in mice. Development.

[31] Vaziri H, Dessain SK, Eaton EN, Imai SI, Frye RA, Pandita TK (2001). hSIR2SIRT1 functions as an NAD-dependent p53 deacetylase. Cell.

[32] González-Sancho JM, Aguilera O, García JM, Pendás-Franco N, Peña C, Cal S (2005). The Wnt antagonist DICKKOPF-1 gene is a downstream target of β-catenin/TCF and is downregulated in human colon cancer. Oncogene.

[33] Aguilera O, Peña C, García JM, Larriba MJ, Ordóñez-morán P, Navarro D (2007). The Wnt antagonist DICKKOPF-1 gene is induced by 1α,25-dihydroxyvitamin D3 associated to the differentiation of human colon cancer cells. Carcinogenesis.

[34] Network TCGA (2012). Comprehensive molecular portraits of human breast tumors. Nature.

[35] Yaeger R, Chatila WK, Lipsyc MD, Hechtman JF, Cercek A, Sanchez-Vega F (2018). Clinical Sequencing Defines the Genomic Landscape of Metastatic Colorectal Cancer. Cancer Cell.

[36] Merlos-Suárez A, Barriga FM, Jung P, Iglesias M, Céspedes MV, Rossell D (2011). The intestinal stem cell signature identifies colorectal cancer stem cells and predicts disease relapse. Cell Stem Cell.

[37] Pálmer HG, González-Sancho JM, Espada J, Berciano MT, Puig I, Baulida J (2001). Vitamin D3 promotes the differentiation of colon carcinoma cells by the induction of E-cadherin and the inhibition of β-catenin signaling. Journal of Cell Biology.

[38] Beildeck ME, Islam M, Shah S, Welsh JE, Byers SW (2009). Control of TCF-4 expression by VDR and vitamin D in the mouse mammary gland and colorectal cancer cell lines. PLoS One.

[39] Glozak MA, Sengupta N, Zhang X, Seto E (2005). Acetylation and deacetylation of non-histone proteins. Gene.

[40] Yang XJ, Seto E (2008). Lysine acetylation: codified crosstalk with other posttranslational modifications. Mol Cell.

[41] Xiong Y, Guan KL (2012). Mechanistic insights into the regulation of metabolic enzymes by acetylation. Journal of Cell Biology.

[42] Sabir MS, Khan Z, Hu C, Galligan MA, Dussik CM, Mallick S (2017). SIRT1 enzymatically potentiates 1,25-dihydroxyvitamin D3 signaling via vitamin D receptor deacetylation. J Steroid Biochem Mol Biol.

[43] White JH, Sarmadi F, Artusa P (2024). The diverse genomic mechanisms of action of the vitamin D receptor. Feldman and Pike' s Vitamin D.

[44] Adorini L (2005). Intervention in autoimmunity: The potential of vitamin D receptor agonists. Cell Immunol.

[45] An B-S, Tavera-Mendoza LE, Dimitrov V, Wang X, Calderon MR, Wang H-J (2010). Stimulation of Sirt1-Regulated FoxO Protein Function by the Ligand-Bound Vitamin D Receptor. Mol Cell Biol.

[46] Iwahara N, Hisahara S, Hayashi T, Horio Y (2009). Transcriptional activation of NAD+-dependent protein deacetylase SIRT1 by nuclear receptor TLX. Biochem Biophys Res Commun.

[47] Liarte S, Alonso-Romero JL, Nicolás FJ (2018). SIRT1 and Estrogen Signaling Cooperation for Breast Cancer Onset and Progression. Front Endocrinol (Lausanne).

[48] Longo VD, Kennedy BK (2006). Sirtuins in Aging and Age-Related Disease. Cell.

[49] Brooks CL, Gu W (2009). How does SIRT1 affect metabolism, senescence and cancer?. Nat Rev Cancer.

[50] Liu T, Liu PY, Marshall GM (2009). The critical role of the class III histone deacetylase SIRT1 in cancer. Cancer Res.

[51] Wang RH, Sengupta K, Li C, Kim HS, Cao L, Xiao C (2008). Impaired DNA Damage Response, Genome Instability, and Tumorigenesis in SIRT1 Mutant Mice. Cancer Cell.

[52] Firestein R, Blander G, Michan S, Oberdoerffer P, Ogino S, Campbell J (2008). The SIRT1 Deacetylase Suppresses Intestinal Tumorigenesis and Colon Cancer Growth. PLoS One.

[53] Chen X, Sun K, Jiao S, Cai N, Zhao X, Zou H (2014). High levels of SIRT1 expression enhance tumorigenesis and associate with a poor prognosis of colorectal carcinoma patients. Sci Rep.

[54] Wei Z, Xia J, Li J, Cai J, Shan J, Zhang C (2023). SIRT1 promotes glucolipid metabolic conversion to facilitate tumor development in colorectal carcinoma. Int J Biol Sci.

[55] Yu X, Li Z, Bai R, Tang F (2022). Transcriptional factor 3 binds to sirtuin 1 to activate the Wnt/β-catenin signaling in cervical cancer. Bioengineered.

[56] Ge X, Jin Q, Zhang F, Yan T, Zhai Q (2009). PCAF Acetylates-Catenin and Improves Its Stability. Mol Biol Cell.

[57] Wolf D, Rodova M, Miska EA, Calvet JP, Kouzarides T (2002). Acetylation of beta-catenin by CREB-binding protein (CBP). J Biol Chem.

[58] Lévy L, Wei Y, Labalette C, Wu Y, Renard C-A, Buendia MA (2004). Acetylation of-Catenin by p300 Regulates-Catenin-Tcf4 Interaction. Mol Cell Biol.

[59] You H, Li Q, Kong D, Liu X, Kong F, Zheng K (2022). The interaction of canonical Wnt/β-catenin signaling with protein lysine acetylation. Cell Mol Biol Lett.

[60] Holloway KR, Calhoun TN, Saxena M, Metoyer CF, Kandler EF, Rivera CA (2010). SIRT1 regulates Dishevelled proteins and promotes transient and constitutive Wnt signaling. Proc Natl Acad Sci U S A.

[61] Zhou Y, Zhou Z, Zhang W, Hu X, Wei H, Peng J (2015). SIRT1 inhibits adipogenesis and promotes myogenic differentiation in C3H10T1/2 pluripotent cells by regulating Wnt signaling. Cell Biosci.

[62] Liu E, Zhou Q, Xie A-J, Li X, Li M, Ye J (2020). Tau acetylates and stabilizes β-catenin thereby promoting cell survival. EMBO Rep.

[63] Berridge MJ (2017). Vitamin D deficiency and diabetes. Biochemical Journal.

[64] García-Martínez JMM, Chocarro-Calvo A, Moya CMM, García-Jiménez C (2009). WNT/β-catenin increases the production of incretins by entero-endocrine cells. Diabetologia.

[65] García-Jiménez C, Gutiérrez-Salmerón M, Chocarro-Calvo A, García-Martinez JM, Castaño A, De La Vieja A (2016). From obesity to diabetes and cancer: Epidemiological links and role of therapies. Br J Cancer.

[66] Gutiérrez-Salmerón M, Lucena SR, Chocarro-Calvo A, García-Martínez JM, Martín Orozco RM, García-Jiménez C (2021). Metabolic and hormonal remodeling of colorectal cancer cell signalling by diabetes. Endocr Relat Cancer.

[67] Gutiérrez-Salmerón M, Lucena SR, Chocarro-Calvo A, García-Martínez JM, Martín Orozco RM, García-Jiménez C (2021). Remodelling of colorectal cancer cell signalling by microbiota and immunity in diabetes. Endocr Relat Cancer.

[68] Ghareghomi S, Arghavani P, Mahdavi M, Khatibi A, García-Jiménez C, Moosavi-Movahedi AA (2024). Hyperglycemia-driven signaling bridges between diabetes and cancer. Biochem Pharmacol.

[69] Chen Y, Chen M, Deng K (2023). Blocking the Wnt/β-catenin signaling pathway to treat colorectal cancer: Strategies to improve current therapies (Review). Int J Oncol.

[70] Groenewald W, Lund AH, Gay DM (2023). The Role of WNT Pathway Mutations in Cancer Development and an Overview of Therapeutic Options. Cells.

[71] Ali A, Shah SA, Zaman N, Uddin MN, Khan W, Ali A (2021). Vitamin D exerts neuroprotection via SIRT1/nrf-2/ NF-kB signaling pathways against D-galactose-induced memory impairment in adult mice. Neurochem Int.

[72] Strycharz J, Rygielska Z, Swiderska E, Drzewoski J, Szemraj J, Szmigiero L (2018). SIRT1 as a Therapeutic Target in Diabetic Complications. Curr Med Chem.

