# Supplementary material for: SIRT1 Mediates the Antagonism of Wnt/β-Catenin Pathway by Vitamin D in Colon Carcinoma Cells
Source: Int J Biol Sci. 2024 Oct 7;20(14):5495–509. doi: 10.7150/ijbs.95875 (PMC11528448; doi:10.7150/ijbs.95875)
Supplement: Supplementary file 1 — Supplementary figures and tables. [file ijbsv20p5495s1.pdf]

**Supplementary information**

**Supplementary Table ST1:** Key resources.

**Supplementary Table ST2:** Clinicopathological characteristics of CRC patients included in the study.

**Supplementary Figure S1: 1,25(OH)$_2$D$_3$ opposes Wnt-induced β-catenin nuclear accumulation and signalling in carcinoma cells.**

(S1A) Confocal immunostaining imaging of the increase in nuclear SIRT1 content in response to 1,25(OH)$_2$D$_3$ (100 nM, 24 h) in HCT 116 cells. Images corresponding to single nuclear staining using DAPI and to merged DAPI + anti-SIRT1 staining are shown. Scale bars: 25 μm.

(S1B) Western blot analysis of SIRT expression in human cell lines from pancreatic cancer (Panc-1, PL-45) and melanoma (A-375, 501mel) treated with 1,25(OH)$_2$D$_3$ (100 nM) for 24 h. TBP and Lamin B1 were used as loading controls.

(S1C) 1,25(OH)$_2$D$_3$ reduces the level of nuclear β-catenin lysine acetylation. Nuclear extracts (NE) from HCT 116 or HT-29 CRC cells were immunoprecipitated (IP) using anti-β-catenin antibodies and analysed by western blotting using anti-acetyl-lysine antibody. TBP in the flow through (FT) was used as control.

(S1D) Representative western blots and statistical analysis of 1,25(OH)$_2$D$_3$ effects on the nuclear β-catenin content in HCT 116 and HT-29 cells. TBP was used as loading control. The full distribution of the data from at least 3 independent experiments is shown. Mean ± SEM of replicates is displayed to illustrate the representability of the western blot shown.

(S1E) Left, confocal immunostaining imaging of nuclear β-catenin in HCT 116 cells treated with Wnt3A in the presence or absence of 1,25(OH)$_2$D$_3$ for 24 h. Scale bars: 25 μm. Right, quantification of fluorescence intensity in 3 independent experiments (2-3 fields/each) using ImageJ software. Mean ± SD is displayed to show variability.

(S1F) RT-qPCR analysis of *MYC* RNA and *CCND1* RNA levels in HCT 116 cells treated Wnt3A (100 ng/ml) in the presence or absence of 1,25(OH)$_2$D$_3$ (100 nM) for 24 h. Values were normalized to those of 18S rRNA and are presented as Mean ±SD.

(S1G) Western blot analysis of the levels of SIRT7 and HDAC-1 in nuclear extracts (NE) of HCT 116 cells treated with 1,25(OH)$_2$D$_3$ (100 nM) or vehicle for 24 h.

(S1H) Effect of pre- and post-treatment with 1,25(OH)$_2$D$_3$ on the high nuclear content of β-catenin protein induced by LiCl-mediated Wnt signalling. Western blot of β-catenin protein in nuclear extracts (NE) of HCT 116 cells. Lamin A/C was used as loading control. Left: cells were treated or not with 1,25(OH)$_2$D$_3$ (100 nM) for 24 h and then treated with LiCl (40 mM) for additional 24 h. Right, cells were first treated with LiCl and 24 h later with 1,25(OH)$_2$D$_3$ or vehicle under the same conditions. Mean ± SEM of triplicates is displayed.

Statistical analysis of at least 3 independent experiments by One-Way ANOVA (panel S1D) or Student t-test (panels S1E, S1F and S1H); *$P < 0.05$; **$P < 0.01$; ***$P < 0.001$.

**Supplementary Figure S2: Acetylation of SIRT1 substrates in human colon carcinomas and established cell lines.**

(S2A) - (S2C) Western blot analyses of the activation by 1,25(OH)$_2$D$_3$ of SIRT1 deacetylase activity on several substrates. HCT 116 CRC cells were treated with 1,25(OH)$_2$D$_3$ (100 nM) or vehicle in the presence or absence of the EX527 SIRT1 inhibitor (10 μM) or the SRT1720 SIRT1 activator (10 μM) for 24 h. Levels of AceH3K9 and AceH4 (K5, 8, 12, 16) (S2A) and of p53 in nuclear extracts (NE) (S2B) and of Acep53K382 in whole cell extracts (WCE) (S2C) are shown.

(S2D) Western blot analyses of the level of AceH4 (K5, 8, 12, 16) and of Acep53K382 in nuclear extracts (NE) of HCT 116 cells that were transfected with expression vectors for Myc-tagged WT SIRT1 or the constitutively active K610R SIRT1 mutant or the catalytically dead H363Y SIRT1 mutant, or with an empty vector (-). Expression of the exogenous SIRT1 proteins was analysed using anti-Myc antibody. TBP and Tubulin were used as loading controls.

(S2E) Representative immunostaining micrographs (40X) of consecutive tumour sections from CRC patients using antibodies against SIRT1 (brown) and its substrates β-catenin (brown) and AceH3K9, AceH4 (K5,8,12,16) and Acep53 (K382) (magenta). Two

examples of patients with high expression and two with low expression of SIRT1 are shown. Scale bars, 20 μm.

(S2F) Representative micrographs (10X) to illustrate example cut-offs from immunostaining of CRC patient samples for both nuclear and cytoplasmic β-catenin (brown).

**Supplementary Figure S3: Effect of 1,25(OH)$_2$D$_3$ on the expression of β-catenin and Wnt target genes in the presence of SIRT1 modulators**

(S3A-B) Effect of the general sirtuin inhibitor nicotinamide (NAA) on the effect of 1,25(OH)$_2$D$_3$ on β-catenin protein expression in CRC cells. (S3A) Confocal immunostaining imaging of β-catenin (green) in HCT 116 cells treated with 1,25(OH)$_2$D$_3$ (100 nM) or vehicle in the presence or absence of NAA (300 μM) for 24 h. Lamin B antibody (red) marked the nuclear envelope. (S3B) Western blot analysis of β-catenin protein content in nuclear extracts (NE) of HCT 116 or HT-29 cells treated with 1,25(OH)$_2$D$_3$ or vehicle in the presence or absence of NAA (300 μM) for 24 h. TBP was used as loading control (S3C) Comparison of the efficiency to deplete SIRT1 protein expression of siRNAs from Santa Cruz and Dharmacon. Representative western blot analyses of nuclear extracts (NE) of HCT 116 and HT-29 cells. TBP was used as loading control.

(S3D) Western blot analysis of the effect of SIRT1 depletion (siRNA from Dharmacon) on the nuclear levels of β-catenin in HT-29 CRC cells. TBP served as loading control.

(S3E-F) SIRT1 mediates 1,25(OH)$_2$D$_3$ action on Wnt signalling. RT-qPCR analysis of the regulation of *AXIN 2* (S3E) and *DKK1* (S3F) RNA levels in HCT 116 cells by treatment with 1,25(OH)$_2$D$_3$ (100 nM) in the presence or absence of the EX527 SIRT1 inhibitor (10 μM) or the SRT1720 SIRT1 activator (10 μM) for 24 h.

(S3G-H) SIRT1 mediates 1,25(OH)$_2$D$_3$ action on Wnt target proteins. Western blot analyses of the regulation of Myc and Cyclin D1 proteins in HCT 116 cells by treatment with 1,25(OH)$_2$D$_3$ (100 nM) in the presence or absence of the EX527 SIRT1 inhibitor (10 μM) or the SRT1720 SIRT1 activator (10 μM) for 24 h. TBP was used as loading control.

Mean ± SEM of triplicates is displayed for western blots in panels B, D, G and H. Median ± SD is shown for RT-qPCR in panels E and F. Statistical analysis by One-Way ANOVA

of at least 3 independent experiments is performed; *$P < 0.05$; **$P < 0.01$; ***$P < 0.001$. Specific pairs of variables were additionally compared using Student t-test (#): #$P < 0.05$.

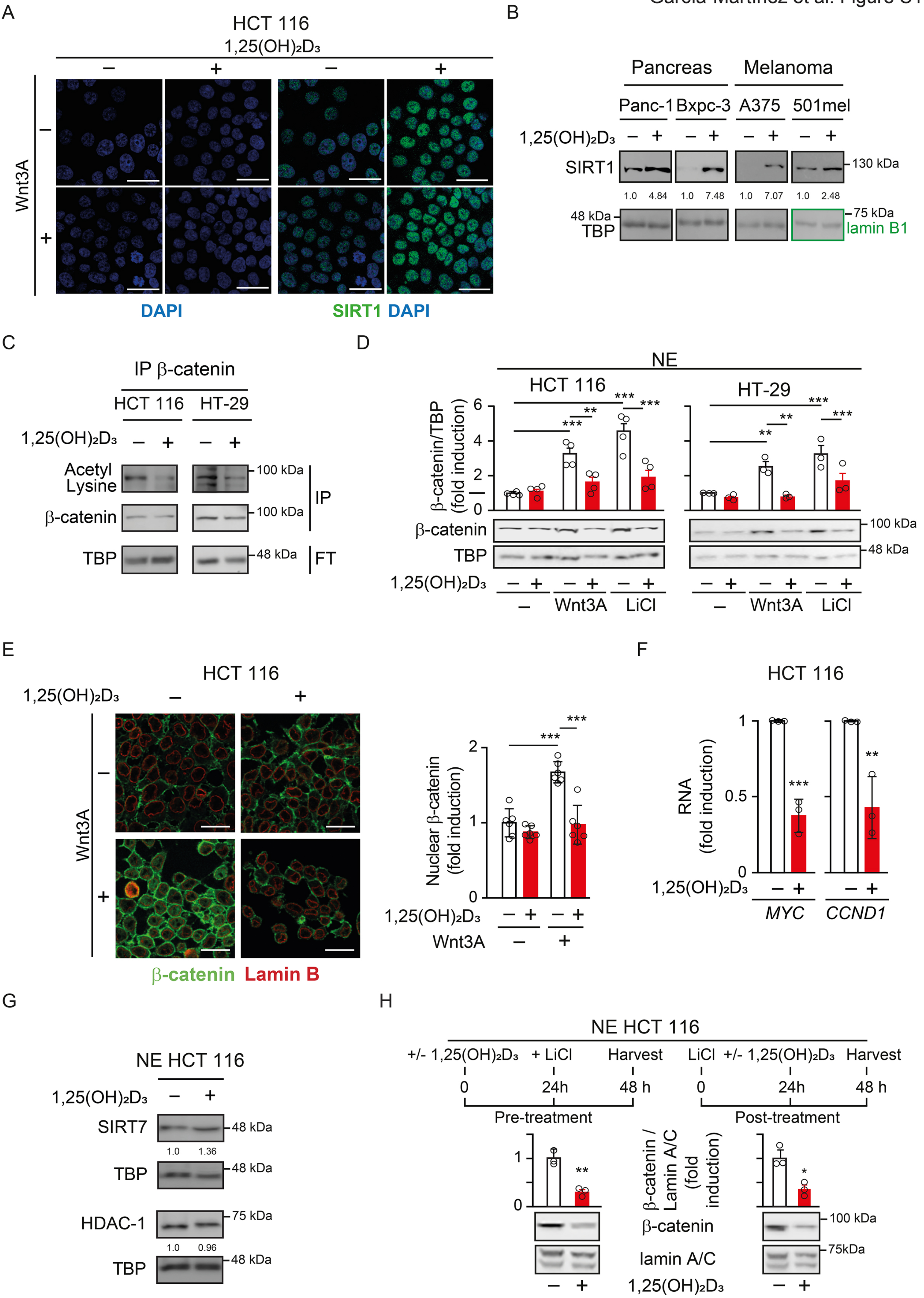

A
HCT 116
1,25(OH)₂D₃
Wnt3A
DAPI
SIRT1 DAPI
B
Pancreas
Melanoma
Panc-1
Bxpc-3
A375
501mel
1,25(OH)₂D₃
SIRT1
130 kDa
1.0 4.84 1.0 7.48 1.0 7.07 1.0 2.48
48 kDa
TBP
75 kDa
lamin B1
C
IP β-catenin
HCT 116
HT-29
1,25(OH)₂D₃
Acetyl Lysine
β-catenin
TBP
100 kDa
100 kDa
48 kDa
IP
FT
D
NE
HCT 116
HT-29
β-catenin/TBP (fold induction)
β-catenin
TBP
100 kDa
48 kDa
1,25(OH)₂D₃
Wnt3A
LiCl
E
HCT 116
1,25(OH)₂D₃
Wnt3A
β-catenin
Lamin B
Nuclear β-catenin (fold induction)
F
HCT 116
RNA (fold induction)
1,25(OH)₂D₃
MYC
CCND1
G
NE HCT 116
1,25(OH)₂D₃
SIRT7
48 kDa
1.0 1.36
TBP
48 kDa
HDAC-1
75 kDa
1.0 0.96
TBP
48 kDa
H
NE HCT 116
+/- 1,25(OH)₂D₃
+ LiCl
Harvest
LiCl
+/- 1,25(OH)₂D₃
Harvest
0
24h
48 h
Pre-treatment
Post-treatment
β-catenin / Lamin A/C (fold induction)
β-catenin
lamin A/C
100 kDa
75kDa
1,25(OH)₂D₃

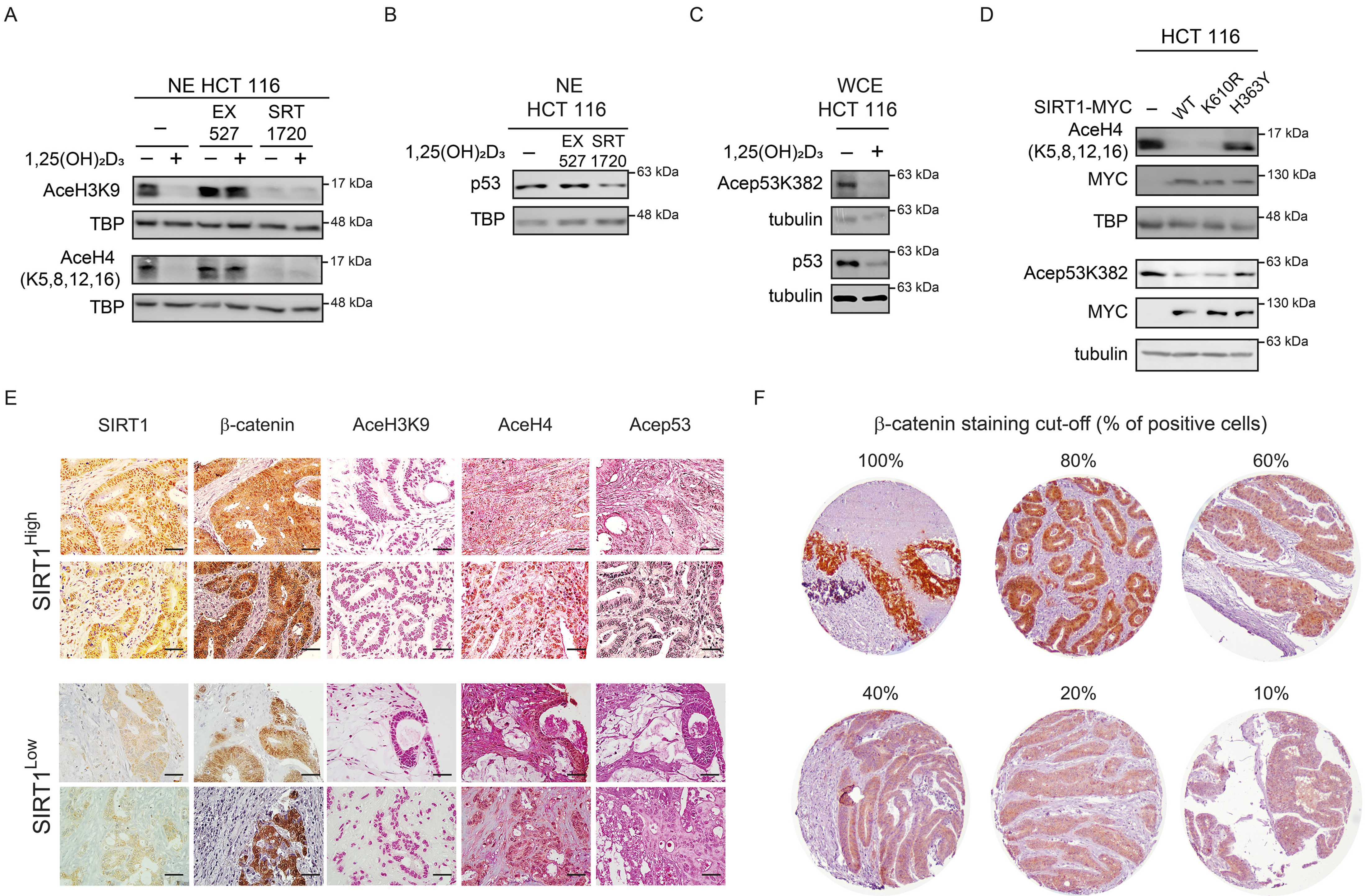
A
NE HCT 116
–
EX 527
SRT 1720
1,25(OH)₂D₃ – + – + – +
AceH3K9
17 kDa
TBP
48 kDa
AceH4 (K5,8,12,16)
17 kDa
TBP
48 kDa
B
NE HCT 116
1,25(OH)₂D₃ – EX 527 SRT 1720
p53
63 kDa
TBP
48 kDa
C
WCE HCT 116
1,25(OH)₂D₃ – +
Acep53K382
63 kDa
tubulin
63 kDa
p53
63 kDa
tubulin
63 kDa
D
HCT 116
SIRT1-MYC – WT K610R H363Y
AceH4 (K5,8,12,16)
17 kDa
MYC
130 kDa
TBP
48 kDa
Acep53K382
63 kDa
MYC
130 kDa
tubulin
63 kDa
E
SIRT1
β-catenin
AceH3K9
AceH4
Acep53
SIRT1High
SIRT1Low
F
β-catenin staining cut-off (% of positive cells)
100%
80%
60%
40%
20%
10%

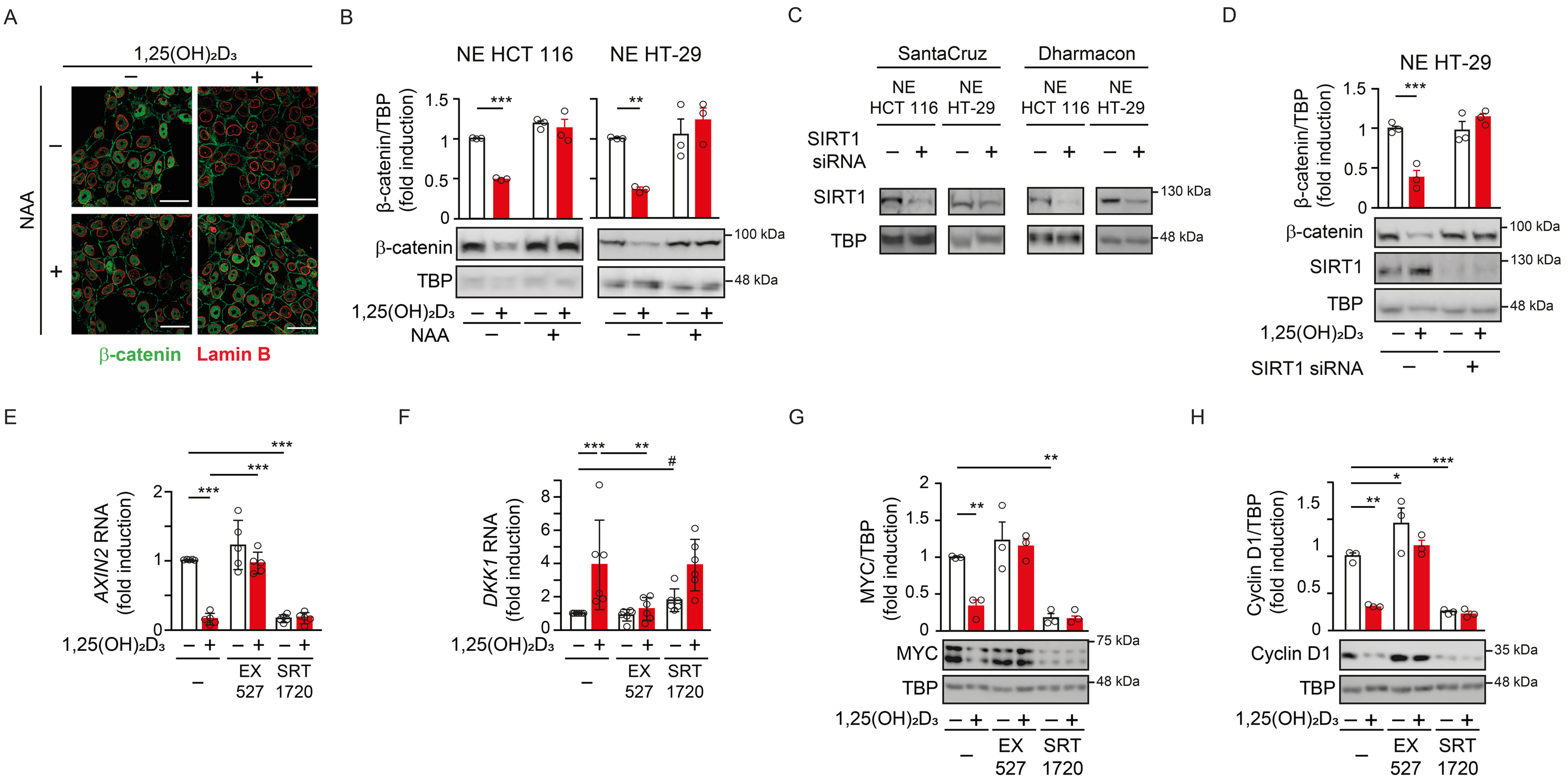

A
1,25(OH)2D3
NAA
β-catenin Lamin B
B
NE HCT 116
NE HT-29
β-catenin/TBP (fold induction)
β-catenin
TBP
100 kDa
48 kDa
1,25(OH)2D3
NAA
C
SantaCruz
Dharmacon
NE HCT 116
NE HT-29
SIRT1 siRNA
SIRT1
TBP
130 kDa
48 kDa
D
NE HT-29
β-catenin/TBP (fold induction)
β-catenin
SIRT1
TBP
100 kDa
130 kDa
48 kDa
1,25(OH)2D3
SIRT1 siRNA
E
AXIN2 RNA (fold induction)
1,25(OH)2D3
EX 527
SRT 1720
F
DKK1 RNA (fold induction)
1,25(OH)2D3
EX 527
SRT 1720
G
MYC/TBP (fold induction)
MYC
TBP
75 kDa
48 kDa
1,25(OH)2D3
EX 527
SRT 1720
H
Cyclin D1/TBP (fold induction)
Cyclin D1
TBP
35 kDa
48 kDa
1,25(OH)2D3
EX 527
SRT 1720

**Supplementary Table ST1. Key resources**

| Reagent or Resource | Source | Identifier |
|---|---|---|
| **Antibodies** | | |
| Rabbit Polyclonal anti-Acetylated-Lysine | Cell Signaling | Cat# 9441 |
| Rabbit Polyclonal anti-β-Catenin (C18) | Santa Cruz Biotechnology | Cat# SC-1496-R |
| Mouse monoclonal anti-β-Catenin | BD | Cat# 610154 |
| Rabbit Polyclonal anti-SIRT1 | Santa Cruz Biotechnology | Cat# SC-15404 |
| Rabbit Polyclonal anti-SIRT1 (D1D7) | Cell Signaling | Cat# 9475 |
| Rabbit monoclonal anti-Histone H3K9-Ace (C5B11) | Cell Signaling | Cat # 9649 |
| Rabbit Polyclonal anti-Histone H3 | Cell Signaling | Cat # 9715 |
| Rabbit Polyclonal anti-Vitamin $D_3$ receptor (D2K6W) | Cell Signaling | Cat #12550 |
| Rabbit Polyclonal anti-MYC (N-262) | Santa Cruz Biotechnology | Cat# SC-764 |
| Mouse monoclonal anti-Cyclin D1 (R-124): | Santa Cruz Biotechnology | Cat# SC-6281 |
| Rabbit monoclonal anti-TBP (D5C9H) | Cell Signaling | Cat#44059 |
| Rabbit Polyclonal anti-Histone H4K5,8,12,16-Ace | Millipore | Cat# 06-598 |
| Rabbit monoclonal anti-K382p53 (10 H13L14) | Thermo Scientific | Cat# 701270 |
| Mouse monoclonal anti-p53 (DO-1) | Santa Cruz Biotechnology | Cat# sc-126 |
| Goat Polyclonal anti-Lamin B (C20) | Santa Cruz Biotechnology | Cat# sc-6216 |
| Mouse monoclonal anti-Lamin A/C (4C11) | Cell Signaling | Cat# 4777 |
| Mouse Monoclonal anti-MYC (9E10) | Santa Cruz Biotechnology | Cat# SC-40 |
| Mouse Monoclonal anti-Tubulin B-5-1-2 | Sigma-Aldrich | Cat# T5168 |
| Mouse Monoclonal anti-SIRT7 (C-3) | Santa Cruz Biotechnology | Cat# sc- 365344 |
| Rabbit Polyclonal anti-HDAC-1 | Upstate | Cat# 06-720 |
| Goat Anti-Rabbit IgG (H+L) HRPO | BIO-RAD | Cat #170-6515 |

| | | |
|---|---|---|
| Goat Anti-Mouse IgG (H+L) HRPO | BIO-RAD | Cat #170-6516 |
| Rabbit-Anti Goat IgG (H+L) HRPO | BIO-RAD | Cat #172-1034 |
| Donkey Anti-Rabbit AlexaFluor488 | Invitrogen | Cat# A21206 |
| Donkey Anti-Mouse AlexaFluor488 | Invitrogen | Cat# A21202 |
| Donkey Anti-Goat AlexaFluor647 | Invitrogen | Cat# A21447 |
| **Bacterial and Virus Strains** | | |
| E. coli-5α | New England Biolabs | NEB5α |
| **Chemicals, Peptides, and Recombinant Proteins** | | |
| 1α,25-Dihydroxyvitamin $D_3$ | Sigma-Aldrich | 17936 |
| Lithium Chloride | Sigma-Aldrich | Cat# L9650 |
| Recombinant Murine Wnt3A | Peprotech | Cat# 315-20 |
| Glucose | Sigma-Aldrich | G8769 |
| BSA | Sigma-Aldrich | A7906 |
| Nicotinamide (NAA) | Sigma-Aldrich | N3376 |
| SRT1720 | Selleckchem | S1129 |
| TRIzol reagent | Invitrogen | Cat#15596026 |
| COMPLETE Protease inhibitor Cocktail | Roche | Cat#04693132001 |
| 7-AAD | Santa Cruz Biotechnology | SC-221210 |
| DAPI (4',6-diamidino-2-fenilindol, dilactato) | Invitrogen | 10184322 |
| 3-(4,5-dimetiltiazol-2-il)-2,5-difeniltetrazol (MTT) | Sigma-Aldrich | Cat# M5655 |
| DMEM medium | Lonza | Cat# 12-604F |
| Bovine Fetal Serum | Sigma | Cat# F7524 |
| JetPEI PolyPlus reagent | Genycell Biotech | Cat# 101-10N |
| JetPRIME PolyPlus reagent | Genycell Biotech | Cat # 114-01 |

| | | |
|---|---|---|
| DYNAbeads Protein A | Invitrogen | Ref 10002D |
| DYNAbeads Protein G | Invitrogen | Ref 10004D |
| **Experimental Models: Cell Lines** | | |
| HCT 116 (Male) colorectal carcinoma | ATCC | Cat# CCL-247, RRID:CVCL_0291 |
| HT-29 (Female) rectosigmoid adenocarcinoma | ATCC | Cat# HTB–38, RRID:CVCL_0320 |
| HCT 116 ShControl | In house | PMID: 21858154 |
| HCT 116 ShVDR | In house | PMID: 21858154 |
| PANC-1 Pancreas Epithelioid Carcinoma. | ATCC | Cat# CRL-1469 |
| PL-45. Pancreatic Ductal Adenocarcinoma | ATCC | Cat# CRL-2558 |
| A375M (Male). Human melanoma cell line | ATCC | Cat# CVCL_2765 |
| 501mel (Female). Human melanoma cell line | Obtained from Ruth Halaban, Yale | (Zakut et al., 1993) |
| **Oligonucleotides** | | |
| Human *SIRT1* K610R mutagenesis primers<br>F 5´-3´: GGTTCTAGTACTGGGGAGAGGAATG AAAGAACTT CAGTGG<br>R 5´-3´: CCAGCCACTGAAGTTCTTTCATTCCTC TCCCCAGT ACTAG | Sigma | This study |
| Human CYCLIN D1 (*CCND1*) qPCR primers<br>F 5´-3´: AAGATCGTCGCCACCTGG<br>R 5´-3´: GGAAGACCTCCTCCTCGCAC | Sigma | This study |
| Human *MYC* qPCR primers<br>F 5´-3´: CTTCTCTCCGTCCTCGGATTCT<br>R 5´-3´: GAAGGTGATCCAGACTCTGACCTT | Sigma | This study |
| Human 18s *rRNA* qPCR primers<br>F 5´-3´: AGTCCCTGCCCTTTGTACACA<br>R 5´-3´: GCCTCACTAAACCATCCAATCG | Sigma | This study |
| Human *DKK1* TaqMan® probe | Applied Biosystems | Hs00167999_m1 |
| Human *AXIN2* TaqMan® probe | Applied Biosystems | Hs00610344_m1 |

| | | |
|---|---|---|
| Sirt1 siRNA | Santa Cruz Biotechnology | Cat# sc-45313 |
| ON-TARGETplus Human SIRT1 (23411) siRNA | Dharmacon | Cat# SO-2917221G |
| siRNA Control | Qiagen | Cat# 1027281 |
| **Recombinant DNA** | | |
| pcDNA3.1-SIRT1-Myc-His | Gift from Prof. Colin R Goding | N/A |
| pcDNA3.1-SIRT1H363Y- Myc-His | Gift from Prof. Colin R Goding | N/A |
| pcDNA3.1-SIRT1 K601R- Myc-His | This study | N/A |
| **Software and Algorithms** | | |
| LAS AF software | Leica | SP5 |
| 3730xl Analyzer | Applied Biosystem | ABI 3730XL |
| GraphPad Prism software | GraphPad Software | https://www.graphpad.com |
| Typhoon scanner control 3.0 | Applied Biosystem | Typhoon 9210 |
| ImageLab | Bio-Rad | ChemiDoc XRS+ System |
| CXP software | Becton-Dickinson | FACSCalibur |
| ImageJ software | https://imagej.nih.gov/ij/download.html | N/A |
| SPSS Statistics version 26 | IBM | N/A |
| The Human Protein Atlas | https://www.proteinatlas.org/ENSG00000096717-SIRT1/pathology/colorectal+cancer | N/A |

**Supplementary Table ST2. Clinicopathological characteristics of CRC patients included in the study.**

| Patient characteristics | Number (%) | |
|---|---|---|
| | Stage II | Stage IV |
| Gender | | |
| Male | 57 (60%) | 27 (48%) |
| Female | 38 (40%) | 23 (41%) |
| N/A | 0 | 6 (11%) |
| Tumor Location | | |
| Cecum | 13 (14%) | 3 ( 5%) |
| Right | 25 (26%) | 10 (18%) |
| Transverse | 7 ( 8%) | 3 ( 5%) |
| Left | 5 ( 5%) | 2 ( 4%) |
| Sigma | 24 (25%) | 21 (38%) |
| Rectum | 21 (21%) | 12 (21%) |
| N/A | 0 | 5 ( 9%) |
| Grade Primary Tumor | | |
| Well differentiated | 18 (19%) | 15 (27%) |
| Moderately differentiated | 69 (73%) | 27 (48%) |
| Poorly differentiated | 8 ( 8%) | 5 ( 9%) |
| N/A | 0 | 9 (16%) |
| Paired Liver Metastasis | | 54 (96%) |
| N/A | | 2 ( 4%) |
| Grade Metastatic Tumor | | |
| Well differentiated | | 12 (21%) |
| Moderately differentiated | | 29 (52%) |
| Poorly differentiated | | 4 ( 7%) |
| N/A | | 11 (20%) |
| Metastasis at diagnosis | | |
| Synchronous | | 35 (62%) |
| Metachronous | | 21 (38%) |
| Pt | | |
| T1 | 3 ( 3%) | 2 ( 4%) |
| T2 | 30 (32%) | 5 ( 9%) |
| T3 | 61 (64%) | 42 (75%) |
| T4 | 1 ( 1%) | 4 ( 7%) |
| N/A | | 3 ( 5%) |
| pN | | |
| N0 | 95 (100%) | 27 (48%) |
| N1 | | 16 (29%) |
| N2 | | 9 (16%) |
| N3 | | 1 ( 2%) |
| N/A | | 3 ( 5%) |
